# The Subiculum: A Potential Site of Ictogenesis in a Neonatal Seizure Model

**DOI:** 10.3389/fneur.2017.00147

**Published:** 2017-04-20

**Authors:** Xin-Xin Wang, Yong-Hua Li, Hai-Qing Gong, Pei-Ji Liang, Pu-Ming Zhang, Qin-Chi Lu

**Affiliations:** ^1^Department of Neurology, Ren Ji Hospital, School of Medicine, Shanghai Jiao Tong University, Shanghai, China; ^2^School of Biomedical Engineering, Shanghai Jiao Tong University, Shanghai, China

**Keywords:** subiculum, hippocampus proper, neonatal seizures, Na^+^–K^+^–2Cl^−^ cotransporter 1, *N*-methyl-d-aspartate receptors

## Abstract

Studies have reported that the subiculum is one origin of interictal-like discharges in adult patients with temporal lobe epilepsy; however, whether the subiculum represents a site of ictogenesis for neonatal seizures remains unclear. In this study, multi-electrode recording techniques were used to record epileptiform discharges induced by low-Mg^2+^ or high-K^+^ artificial cerebrospinal fluid in neonatal mouse hippocampal slices, and the spatiotemporal dynamics of the epileptiform discharges were analyzed. The Na^+^–K^+^–2Cl^−^ cotransporter 1 (NKCC1) blocker, bumetanide, was applied to test its effect upon epileptiform discharges in low-Mg^2+^ model. The effect of *N*-methyl-d-aspartate receptors (NMDARs) antagonist, d-AP5, upon the epileptiform discharges in high-K^+^ model was examined. We found that the neonatal subiculum not only relayed epileptiform discharges emanating from the hippocampus proper (HP) but also initiated epileptiform discharges (interictal- and ictal-like discharges) independently. The latency to onset of the first epileptiform discharge initiated in the subiculum was similar to that initiated in the HP. Bumetanide efficiently blocked seizures in the neonatal HP, but was less effectively in suppressing seizures initiated in the subiculum. In high-K^+^ model, d-AP5 was more effective in blocking seizures initiated in the subiculum than that initiated in the HP. Furthermore, Western blotting analysis showed that NKCC1 expression was lower in the subiculum than that in the HP, whereas the expression of NMDAR subunits, NR2A and NR2B, was higher in the subiculum than that in the HP. Our results revealed that the subiculum was a potential site of ictogenesis in neonatal seizures and possessed similar seizure susceptibility to the HP. GABAergic excitation resulting from NKCC1 may play a less dominant role during ictogenesis in the subiculum than that in the HP. The subicular ictogenesis may be related to the glutamatergic excitation mediated by NMDARs.

## Introduction

In infants, neonatal seizures are frequent and serious conditions occurring in the first 28 days (1, 2). Neonatal seizures can be caused by a variety of brain injuries, such as hypoxia-ischemia, stroke, intracranial hemorrhage, infection, or central nervous system (CNS) malformations (3). Neonates suffering from seizures show a high risk of early death and a series of dysfunctions in neurological development, such as motor or cognitive disability (4). However, traditional antiepileptic drugs are often inappropriate for the treatment of neonatal seizures, as the underlying mechanisms of neonatal seizures are complicated and are not clearly understood (5). Therefore, it is essential to explore the mechanisms of ictogenesis in neonatal seizures, so that we can develop more efficient treatments.

Some studies have demonstrated that the mechanisms of ictogenesis differ when compared between different regions and ages. On the one hand, the capacities of ictogenesis vary from one brain region to another, since not all brain regions possess pacemaker cells and sufficient number of axon collaterals (6, 7). On the other hand, ictogenesis is related to excitatory action of GABA, whereas the switch of GABAergic action from excitation to inhibition occurs at different ages in different regions (8). Developmental changes in GABAergic action are associated with age- and region-related expression of cation chloride cotransporters (9). Thus, the effect of anticonvulsants is both region-specific and age-dependent (10). Consequently, understanding the mechanisms underlying ictogenesis in different regions during the neonatal period is helpful in the elucidation of potential therapeutic targets for neonatal seizures.

The CA3 of the hippocampus proper (HP) has proved to be one of the potential sites of ictogenesis for neonatal seizures (11) and possesses pacemaker cells and abundant axon collaterals (6). As well as CA3, the subiculum, located between the HP and entorhinal cortex (EC), possesses burst firing cells which serve as pacemaker cells, and features an extensive network of axon collaterals (7, 12, 13). Furthermore, evidence obtained from adult rodent animal models of epilepsy (7, 12), and adult temporal lobe epilepsy (TLE) patients (14, 15), has suggested that the subiculum plays a vital role in the initiation and maintenance of interictal-like discharges (IIDs). However, it is still unknown whether the subiculum is a potential site of ictogenesis for neonatal seizures.

It has been demonstrated that excitatory actions of GABA contribute to the initiation of ictal-like discharges (IDs) in the neonatal CA3 (16). Excitatory action of GABA is determined by an elevated intracellular Cl^−^ concentration and a depolarized Cl^−^ equilibrium potential (E_Cl_) (5, 17). The accumulation of intracellular Cl^−^ depends on the action of Na^+^–K^+^–2Cl^−^ cotransporter 1 (NKCC1) (18). Some experiments have indicated that bumetanide, a blocker of NKCC1, abolishes IDs and depresses IIDs in the neonatal CA3 (11). However, NKCC1 expression is age and region dependent (10, 19). If the neonatal subiculum was able to initiate epileptiform discharges (IDs and IIDs) independently, it is uncertain whether these epileptiform discharges also result from the GABAergic excitation caused by NKCC1.

In our experiments, recurrent seizures in neonatal hippocampal slices were induced by low-Mg^2+^ or high-K^+^ artificial cerebrospinal fluid (ACSF). Both models disrupted the excitation/inhibition (E/I) balance of neuronal network (20, 21), which mimicked the E/I imbalance of seizures *in vivo* (22, 23). The low-Mg^2+^ model of epilepsy developed several decades ago (24, 25), and has been widely used because of the clinical relevance. Previous researches have revealed that Mg^2+^ deficits could cause seizures in humans (26, 27). In addition, in those patients with generalized tonic–clonic seizures, the Mg^2+^ concentration in cerebrospinal fluid or serum is lower (28, 29). Furthermore, in animal models of epilepsy, intravenous infusion of Mg^2+^ can relieve seizures (30) and is used as an antiepileptic therapy, particularly in women with eclampsia (31). Low-Mg^2+^ model mainly abolished the blockade of NR2A/B-containing *N*-methyl-d-aspartate receptors (NMDARs) (32) and increased the excitation of the hippocampal neural network (24). Whether the neonatal subiculum expresses abundant NMDARs and whether the activation of NMDARs would induce epileptiform discharges in the neonatal subiculum have not yet been investigated. If lowering the concentration of extracellular Mg^2+^ would induce epileptiform discharges in the neonatal subiculum, then this will provide a new site to study the mechanisms underlying ictogenesis in models of neonatal seizure.

In the present study, the micro-electrode array (MEA) was used to record epileptiform discharges induced by low-Mg^2+^ or high-K^+^ ACSF in neonatal mouse hippocampal slices. Then, the initiation and propagation of epileptiform discharges in these slices were analyzed. The NKCC1 blocker, bumetanide, was then applied to investigate its effect upon epileptiform discharges in the subiculum and the HP in low-Mg^2+^ model. The effect of NMDARs antagonist, d-AP5, upon the epileptiform discharges in high-K^+^ model was examined. The expression levels of NKCC1, NR2A, and NR2B in the subiculum and the HP were then measured by Western blotting. Our data showed that the subiculum is a potential site of ictogenesis in models of neonatal seizure and that the mechanism underlying ictogenesis in the neonatal subiculum was not identical to that in the HP.

## Animals and Methods

### Animals

Male C57BL/6 mice aged 8–10 days (P8–P10) were used in all experiments. Sixteen different litters were used, and two male mice were randomly chosen in each litter. Mice were housed in a controlled laboratory environment with regular animal chow and water *ad libitum* and were maintained under a 12-h light/dark cycle. We made particular effort to minimize the number of animals used and the potential for animal suffering.

### Slice Preparation

Mice were quickly decapitated under deep isoflurane anesthesia. The intact brain was promptly removed and placed in ice-cold and oxygenated ACSF containing the following constituents (in millimolars per liter): 124.0 NaCl, 3.5 KCl, 1.2 NaH_2_PO_4_, 1.3 MgCl_2_⋅6H_2_O, 2.5 CaCl_2_, 25.0 NaHCO_3_, and 10.0 glucose at pH 7.4 (~300 mOSm/l). Hippocampal slices (400 µm) were then prepared using a Vibratome (Series1000, Sectioning Systems, Vibratome, St. Louis, MO, USA). Intact slices were then incubated at 28°C for at least 2 h prior to use. In this study, only one slice was selected from each mouse, and all slices adopted came from the similar position. The slices included the HP (CA3, CA1, and DG) and the subiculum. Epileptiform discharges were induced by low-Mg^2+^ or high-K^+^ ACSF. The constituents of low-Mg^2+^ ACSF were as follows (in millimolars per liter): 124.0 NaCl, 3.5 KCl, 1.2 NaH_2_PO_4_, 2.5 CaCl_2_, 25.0 NaHCO_3_, and 10.0 glucose at pH 7.4 (~300 mOSm/l). The constituents of high-K^+^ ACSF were as follows (in millimolars per liter): 119.0 NaCl, 8.5 KCl, 1.2 NaH_2_PO_4_, 1.3 MgCl_2_⋅6H_2_O, 2.0 CaCl_2_, 25.0 NaHCO_3_, and 10.0 glucose at pH 7.4 (~300 mOSm/l).

### Chemicals and Drugs

Bumetanide, dimethyl sulfoxide (DMSO), and d(−)-2-amino-5-phosphonopentanoic acid (d-AP5) were purchased from Sigma-Aldrich Co., Ltd. (USA), and the other chemicals were acquired from Sinopharm Chemical Reagent Co., Ltd. (China). Bumetanide was dissolved in DMSO and then added to low-Mg^2+^ ACSF, shortly before the experiment. d-AP5 was dissolved in high-K^+^ ACSF directly before the experiment.

### Electrophysiological Recording

**Figure 1:**
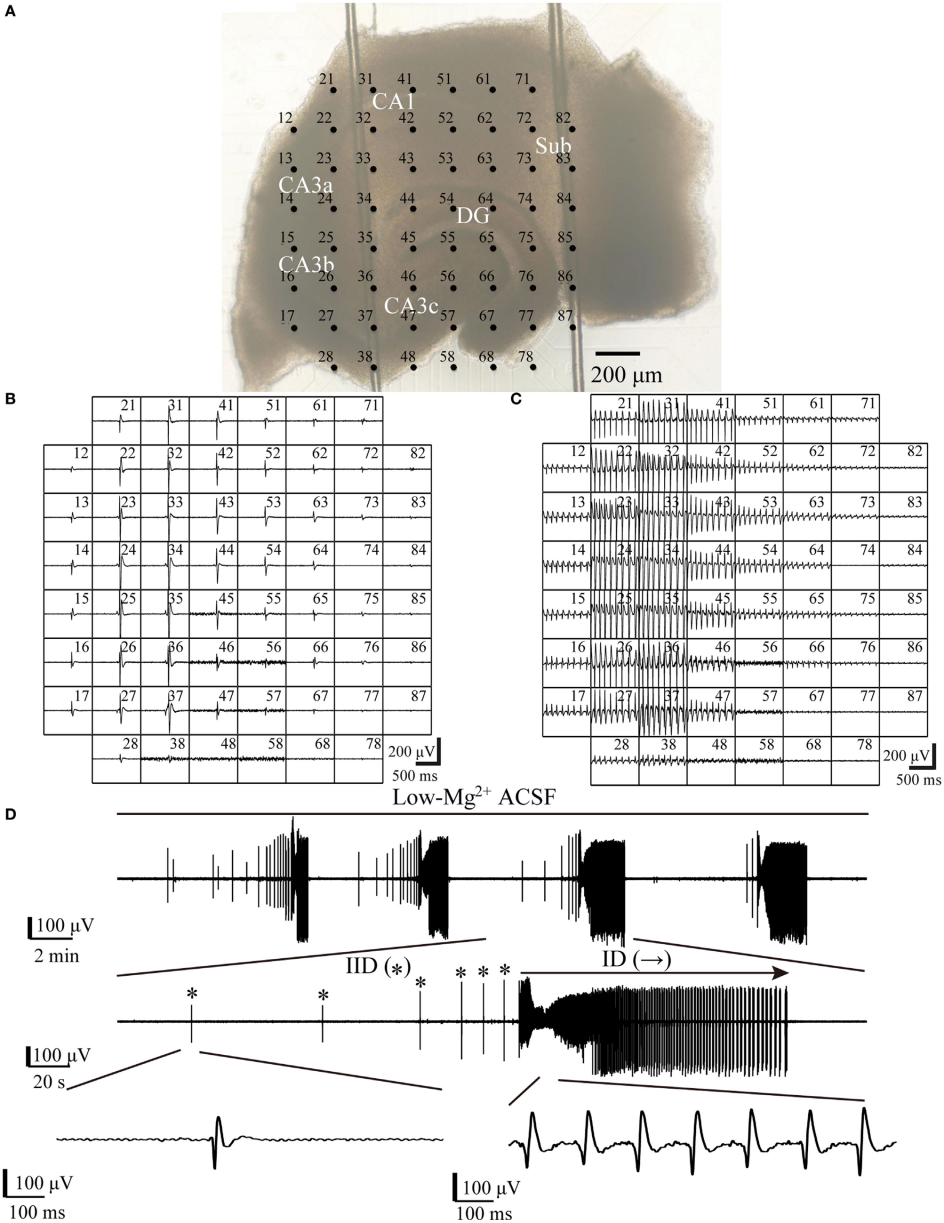
**One example slice and low-Mg^2+^-induced epileptiform discharges**. **(A)** An image of a hippocampal slice mounted on micro-electrode array (MEA). The black dots stand for the electrodes, with electrode number labeled at its top left corner. The slice is composed of the hippocampus proper (DG, CA3a/b/c, CA1) and the subiculum (Sub). **(B,C)** Low-Mg^2+^-induced interictal-like discharges (IIDs) **(B)** and ictal-like discharges (IDs) **(C)** recorded by the MEA. Each window represents the recording from one electrode, with each electrode number labeled at its top right corner. **(D)** The upper trace exhibits a period (−2,100 s) of data recorded by one electrode (number 27) in **(A)**, which represents four discharge cycles in the CA3. The middle trace displays one of the four discharge cycles in the upper trace in an expanded time scale, which consists of six IIDs (*) and one ID (→). The lower trace further shows one IID and the ID in the middle trace in a more expanded time scale.

A multi-electrode recording system (MEA60, Multichannel Systems GmbH, Germany) was applied to record the electrical activities arising from the slices. The MEA consisted of 60 electrodes (30 µm in diameter), which were arranged in an 8 × 8 matrix (the four corners left void) with 200 µm tip-to-tip distances (Figure 1A).

For each tissue, a slice was placed in the center of the MEA in a rapid and careful manner. A nylon mesh was used to immobilize the slice. The MEA with the slice was then transferred to the stage of a microscope (Olympus, Japan) and perfused with oxygenated ACSF at 36°C using a temperature control unit (Thermostat HC-X, Multichannel Systems GmbH, Germany). A peristaltic pump (Ismatec, SA, USA) was used to maintain the flow rate at 2 ml/min. The position of the slice on the MEA was observed under a microscope, and images from the slice were captured with a camera (Olympus, Japan).

All data were recorded with a 60-channel amplifier (single-ended, bandwidth 1 Hz–3.4 kHz, amplification 1,200×, input impedance >10^10^ Ω, output impedance 330 Ω) and sampled at 20 kHz. Data were displayed on a computer screen (Figures 1B,C) and stored for off-line analysis.

### Data Analysis

Data analysis was performed using the MC-Rack (Multichannel Systems GmbH, Germany) and MATLAB (MathWorks, USA). Raw data were filtered into the field potential (FP) by a 1- to 100-Hz band-pass filter and then down-sampled from 20 to 1 kHz. Epileptiform discharges were confirmed when the negative or positive peak of the FPs exceeded the threshold (four times the SD from the mean value of 500 ms baseline, in which no significant epileptiform discharges were observed). The epileptiform discharges induced in the hippocampal slices were divided into IIDs and IDs. IDs were similar to the interictal-spike *in vivo*, they were characterized by brief burst, lasted hundreds of seconds, and represented fast, synchronous potentials in a large number of neurons (33–35). IDs resembled the seizures *in vivo*, they consisted of the ictal–tonic discharges, ictal-clonic discharges, and post-ictal depression, and lasted tens of seconds to several minutes (23, 34, 36). As with previous *in vitro* studies, IIDs lasted a few hundred milliseconds to a couple of seconds, while IDs sustained several seconds to tens of seconds (16, 37). In our experiments, the epileptiform events, which lasted more than 5 s were defined as IDs and the other events were defined as IIDs.

The initiation and propagation of epileptiform discharges were determined by comparing the onset-times of the epileptiform discharges recorded by all electrodes in the hippocampal slice, and the onset-time of the epileptiform discharge was defined as the timing when the FP exceeded the threshold (38, 39). The region where the electrode first recorded the epileptiform discharge was defined as the initiation site. The parameters (duration, frequency, amplitude) of the epileptiform discharges in different sub-regions were calculated for further analysis, and were obtained by averaging data recorded by two adjacent electrodes located along the pyramidal layer (CA3, CA1, Sub) or the granule cell layer (DG) of the slices.

### Western Blotting Analysis

Slices were prepared in the same way as they were used for electrophysiology, then the tissue (without perfusion after the low-Mg^2+^ or high-K^+^ ACSF) were rapidly moved to ice-cold 0.1 M phosphate buffer. Under a microscope (Olympus, Japan), the subiculum and HP were isolated with a sharp blade. Then, the tissues were homogenized on ice in RIPA buffer (Sigma-Aldrich, USA) with complete protease inhibitor cocktail (Roche, Germany) using the auto Tissuelyser-24 (Shanghai Jingxin, China) (2× 30 s). Tissue homogenates were centrifuged (12,000 × *g*, 30 min, 4°C), and the supernatants were finally aliquoted and frozen (−80°C) for subsequent analysis.

The concentration of all protein extracts was determined using the BCA protein assay kit (Sangon Biotech, China). Samples, each containing 100 µg of total protein, were loaded and separated by electrophoresis by 10% SDS-PAGE. Proteins were then electro-transferred to polyvinylidene difluoride (PVDF) membranes (Millipore, USA). Membranes were then blocked in 5% bovine serum albumin (BSA) or 5% non-fatty milk dissolved in 1× TBST (140 mM NaCl, 3 mM KCl, 25 mM Tris, 0.1% Tween-20) and then incubated in primary antibody diluted in 5% BSA or 5% non-fatty milk overnight at 4°C. Antibody dilutions were as follows: 1:100 for NKCC1 (rabbit polyclonal antibody; abcam); 1:500 for NR2A (rabbit polyclonal antibody; Cell Signaling technology, CST); 1:500 for NR2B (rabbit monoclonal antibody; Cell Signaling technology, CST); and 1:5,000 for β-actin (rabbit monoclonal antibody; Cell Signaling technology, CST). The membranes were washed in 1× TBST (3× 10 min) after incubation with primary antibodies, then incubated in horseradish peroxidase-conjugated Goat Anti-Rabbit IgG (1:5,000, HSA003) for 2 h at room temperature, and the secondary antibody was diluted in 5% BSA or 5% non-fatty milk. Membranes were then re-washed with 1× TBST (3× 10 min). Finally, protein bands were detected using the ECL chemiluminescent reagent, and ImageJ (NIH, USA) was used to quantify the intensity of each band. Relative protein levels were determined by the ratio of the band intensity of the target protein to its respective β-actin loading control. Then, each protein level in the subiculum was normalized to the corresponding protein level in the HP (each protein level in the HP was regarded as 1.0).

### Statistical Analysis

Data were expressed as means ± SEM. Statistical analysis was conducted using paired *t*-test or one-way analysis of variance (ANOVA) with a significance level of *P* < 0.05.

## Results

### Epileptiform Discharges Induced by Low-Mg^2+^ ACSF

When hippocampal slices were exposed to low-Mg^2+^ ACSF, epileptiform discharges (IIDs and IDs) were induced in both the HP and the subiculum. One example of a slice mounted on the MEA along with the recorded epileptiform discharges are shown in Figure 1. Each discharge cycle, consisting of 2–20 (8.19 ± 0.87, *n* = 21) IIDs and one ID, recurred after a resting period which lasted 113–504 s (241.23 ± 20.54 s, *n* = 21).

### Initiation and Propagation of the Epileptiform Discharges

**Figure 2:**
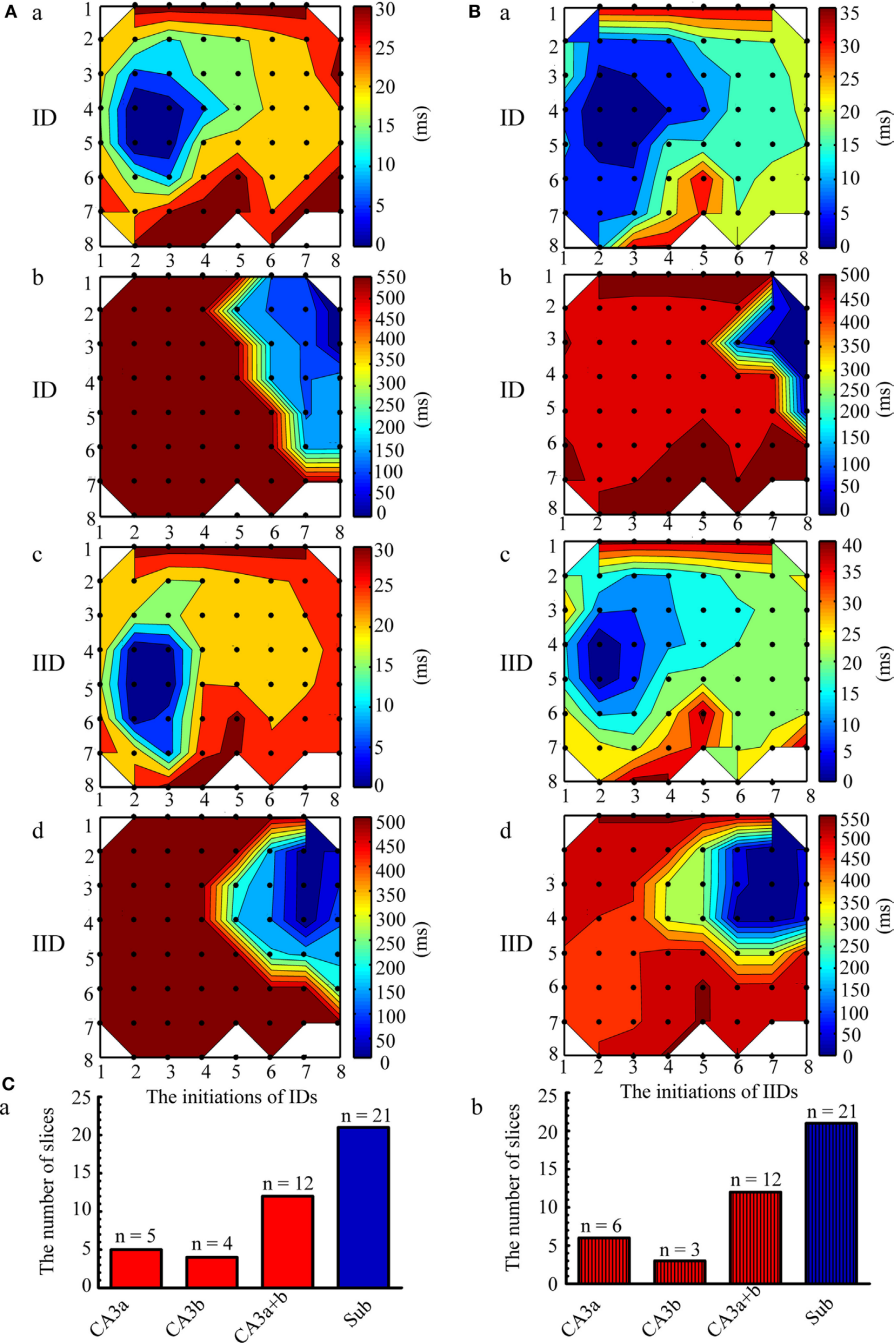
**Initiation and propagation of the ictal-like discharges (IDs) and interictal-like discharges (IIDs)**. **(A)** Examples of the two types of contour plots for the relative onset-time delays of one single ID (a,b) and IID (c,d) in the slice shown in Figure 1A. **(B)** Two types of contour plots for the averaged relative onset-time delays of all the IDs (a,b) and IIDs (c,d) in the slice shown in Figure 1A. The number of the electrodes (black dots) were denoted by the *x* and *y* coordinates. The color bar represents the relative onset-time delays. The different color in different regions symboled the initiate sites and propagation pathways. The epileptiform discharges had two initiations (dark blue regions), CA3a/b (Aa, Ac, Ba, Bc) and the subiculum (Ab, Ad, Bb, Bd). The CA3-origin epileptiform discharges initiated in CA3a/b and propagated bidirectionally to the CA1, subiculum (anterograde), to the CA3c, and DG (retrograde). The Sub-origin epileptiform discharges initiated in the subiculum and did not propagate backward to the CA1. **(C)** The quantitative analysis pertaining to the initiations of the IIDs (a) and IDs (b) in the 21 slices.

The initiation and propagation of IDs and IIDs were analyzed. We calculated the onset-time delays of IDs and IIDs relative to the signals of one electrode and expressed them by contour plots. The averaged relative onset-time delay of all the IDs and IIDs in each slice were also calculated and expressed by contour plots. In each slice, we revealed two initiations and two types of corresponding propagation patterns for IDs. Similar results were also found for IIDs. Figure 2A shows examples of the two types of contour plots for a single epileptiform discharges on the representative slice shown in Figure 1A. Figure 2B shows examples of two types of contour plots for the averaged onset-time delays of all the epileptiform discharges on the representative slice shown in Figure 1A. These data showed that one type of epileptiform discharge initiated in CA3a/b and propagated bidirectionally to the CA1, subiculum (anterograde), CA3c, and DG (retrograde); we defined these as CA3-origin epileptiform discharges. However, the other type initiated in the subiculum, and was defined as Sub-origin epileptiform discharges. As the onset-time delay of the Sub-origin epileptiform discharges between the subiculum and the CA1 exceeded 300 ms, far beyond the time delay shown by epileptiform discharges propagating from the subiculum to the CA1 (12), the Sub-origin epileptiform discharges did not propagate backward to the CA1. The data obtained from the other 20 slices also showed similar results, and the corresponding averaged contour plots in each slice, except the slice shown in Figure 1A, are shown in Figure S1 in Supplementary Material. The quantitative analysis pertaining to the initiations of epileptiform discharges in the 21 hippocampal slices are summarized in Figure 2C. In each slice, the IIDs/IDs had two initiations.

### The CA3- and Sub-Origin Epileptiform Discharges

**Figure 3:**
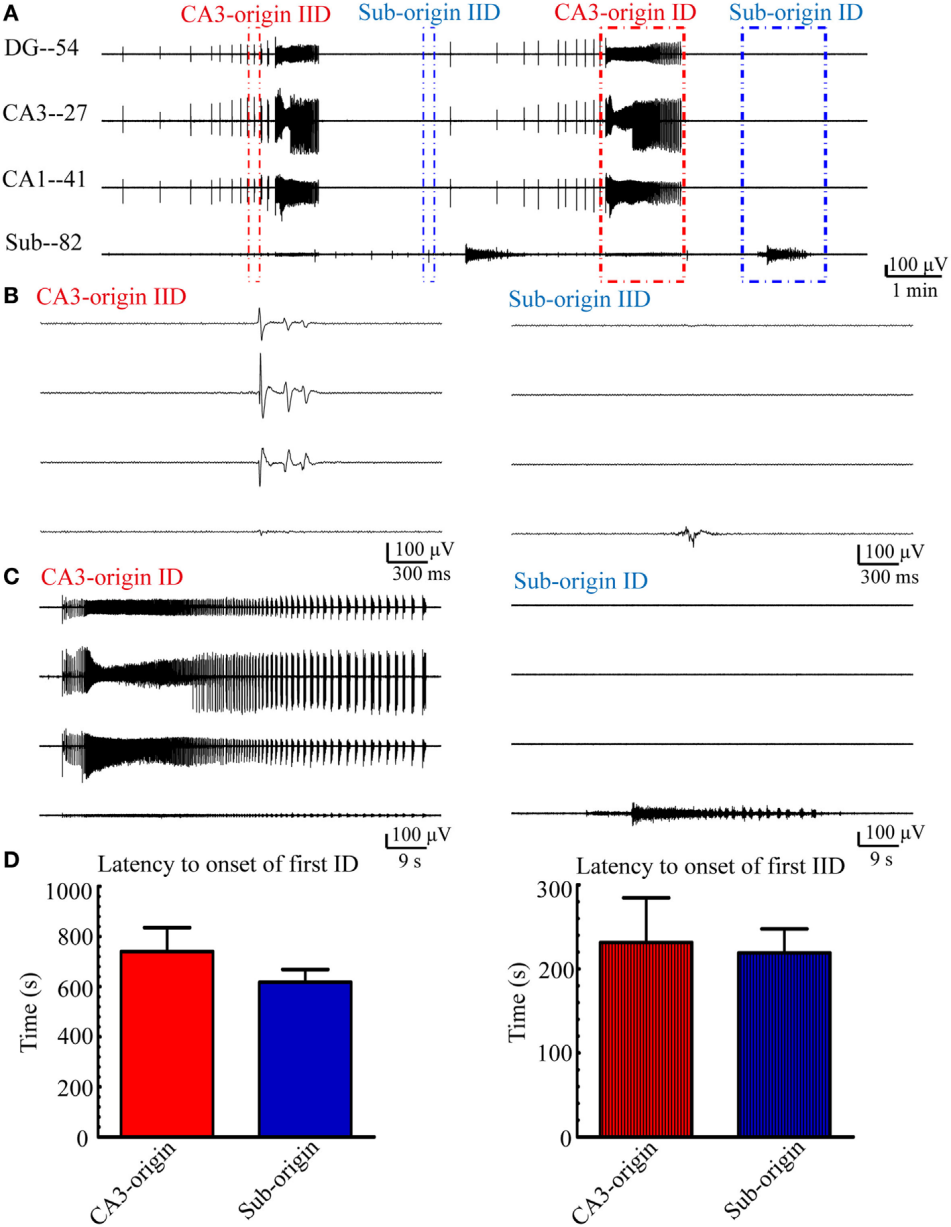
**CA3- and Sub-origin epileptiform discharges**. **(A)** Epileptiform discharges recorded by four electrodes (numbers 54, 27, 41, 82) in the slice shown in Figure 1A, which represents the epileptiform discharges in the DG, CA3, CA1, and subiculum, respectively. The red and blue rectangles represent the CA3- and Sub-origin epileptiform discharges, respectively. **(B)** The interictal-like discharges (IIDs) in rectangles in **(A)** are displayed in an expanded time scale. **(C)** The ictal-like discharges (IDs) in rectangles in **(A)** are shown on an expanded time scale. **(D)** Latency to onset of the first CA3-origin ID/IID in the hippocampus proper and Sub-origin ID/IID in the subiculum. Data were presented as mean ± SEM. There was no significant difference between them (*P* > 0.05, paired *t*-test, *n* = 21).

Examples of the CA3- and Sub-origin epileptiform discharges are illustrated in Figure 3. The CA3-origin epileptiform discharges were recorded in both the HP and the subiculum, whereas the Sub-origin epileptiform discharges were only recorded in the subiculum. After the application of low-Mg^2+^ ACSF, the latency to onset of the first CA3-origin IID was 180–290 s (231.44 ± 53.21 s, *n* = 21), the first Sub-origin IID was 190–250 s (219.07 ± 28.59 s, *n* = 21), the first CA3-origin ID was 230–1,840 s (740.14 ± 95.37 s, *n* = 21), and the first Sub-origin ID was 280–1,540 s (617.64 ± 50.92 s, *n* = 21). There was no significant difference between the latency to onset of the first CA3-origin epileptiform discharge in the HP and the Sub-origin epileptiform discharge in the subiculum. These results revealed that the seizure susceptibility in the subiculum was similar to that in the HP.

**Figure 4:**
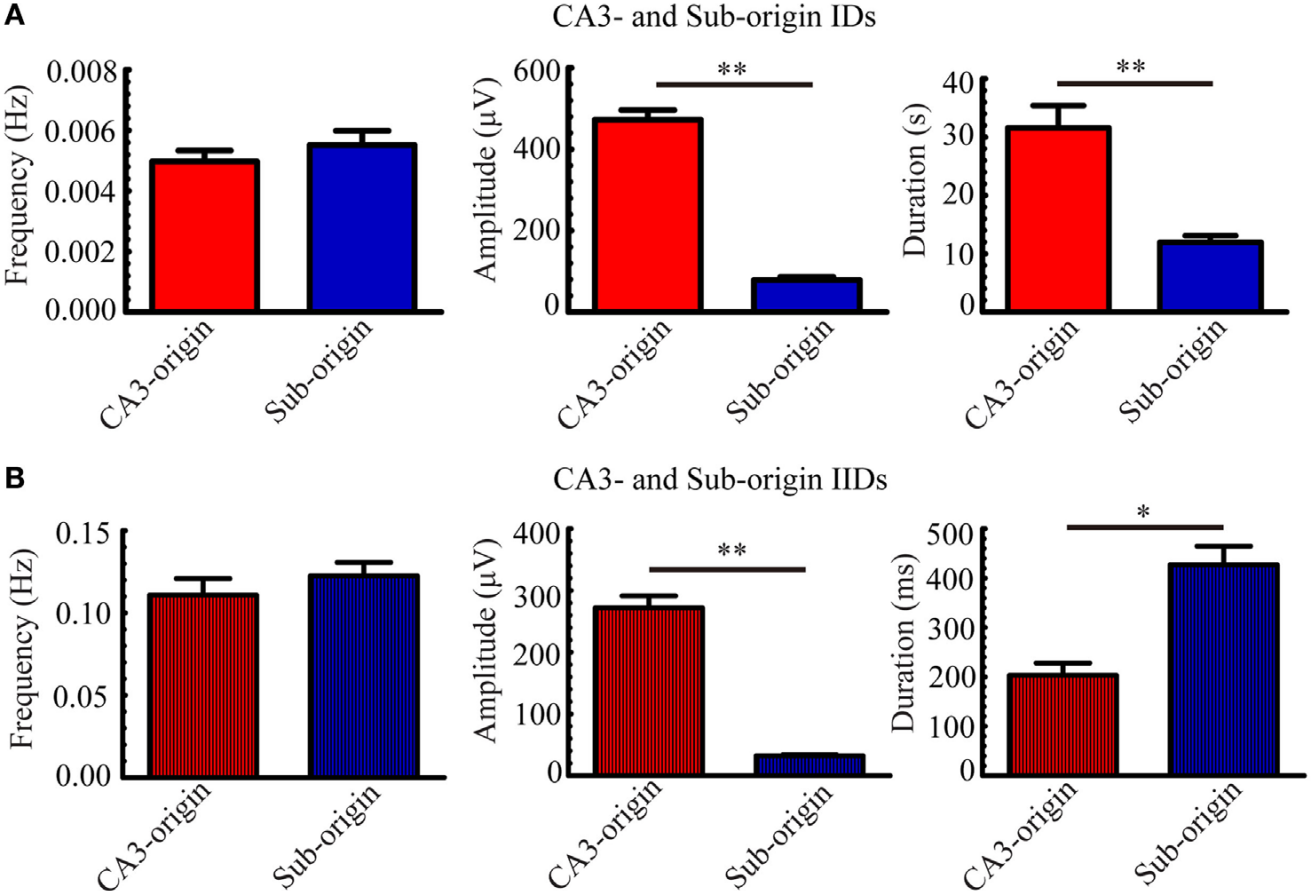
**Parameters of CA3- and Sub-origin ictal-like discharges (IDs)/interictal-like discharge (IIDs)**. **(A,B)** The frequency, amplitude and duration of CA3- and Sub-origin IDs **(A)** and IIDs **(B)**. Data were presented as mean ± SEM. There was no significant difference between the frequencies of CA3- and Sub-origin IDs/IIDs (*P* > 0.05, paired *t*-test, *n* = 21). The amplitude of CA3-origin epileptiform discharges in CA3 was significantly higher than that of the Sub-origin epileptiform discharges in the subiculum (**P < 0.01, paired *t*-test, *n* = 21). The duration of CA3-origin IDs was significantly longer than that of Sub-origin IDs (**P < 0.01, paired *t*-test, *n* = 21). The duration of CA3-origin IIDs was significantly shorter than that of the Sub-origin IIDs (*P < 0.05, paired *t*-test, *n* = 21).

Next, we quantitatively analyzed the parameters (frequency, amplitude, duration) of the epileptiform discharges in our 21 hippocampal slices, and the results are summarized in Figure 4. The frequencies of CA3- and Sub-origin IDs/IIDs were not significantly different (*P* > 0.05, paired *t*-test, *n* = 21). The amplitude of CA3-origin epileptiform discharges in the CA3 was significantly higher than that of the Sub-origin epileptiform discharges in the subiculum (**P < 0.01, paired *t*-test, *n* = 21). The duration of the CA3-origin IDs was significantly longer than that of the Sub-origin IDs (**P < 0.01, paired *t*-test, *n* = 21). The duration of CA3-origin IIDs was significantly shorter than that of the Sub-origin IIDs (*P < 0.05, paired *t*-test, *n* = 21). These results suggested that the CA3- and Sub-origin epileptiform discharges were not identical to each other.

### The Effect of Bumetanide upon Epileptiform Discharges

**Figure 5:**
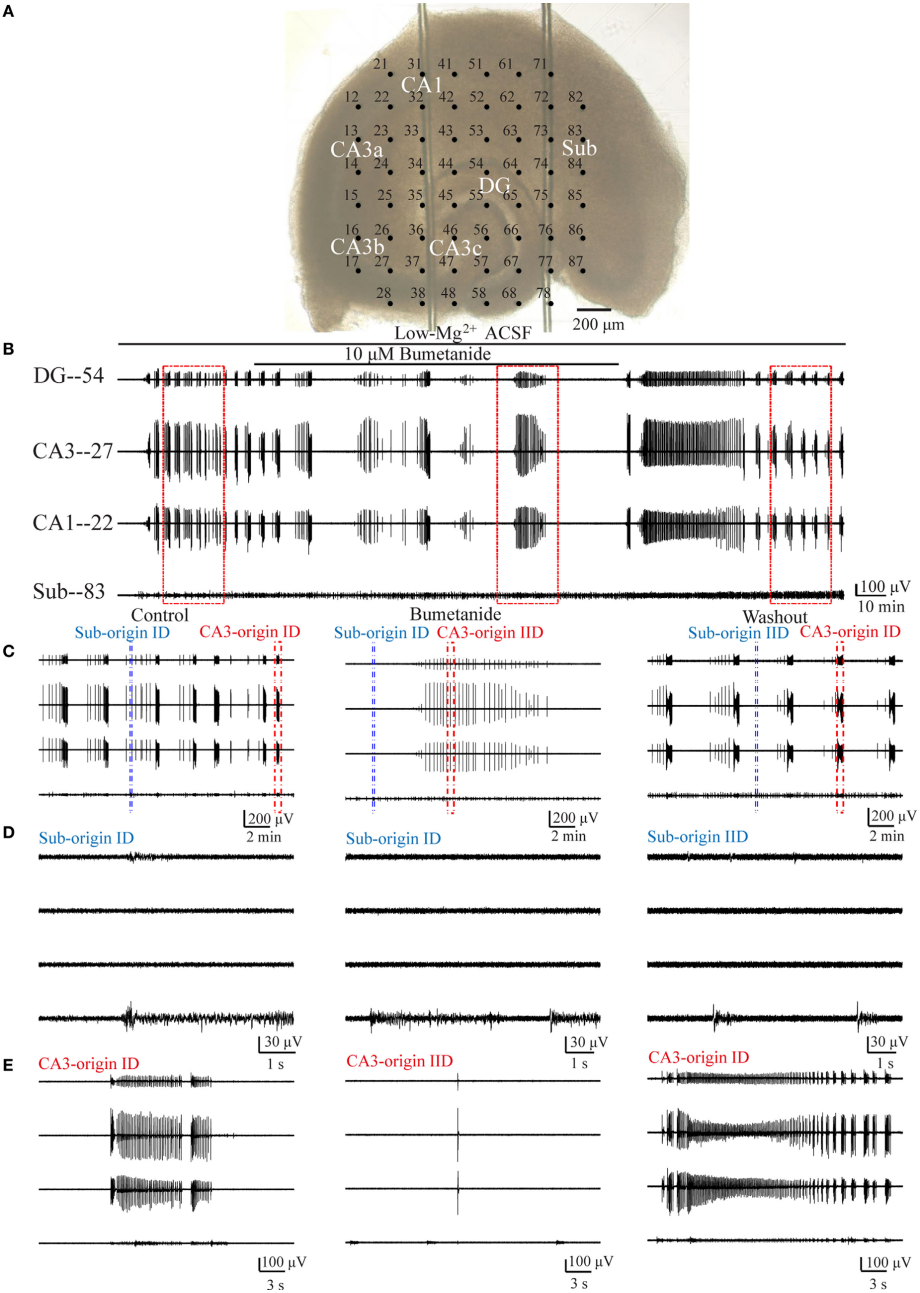
**Effect of bumetanide upon the CA3- and Sub-origin epileptiform discharges**. **(A)** An example slice mounted on micro-electrode array. **(B)** The long-term display of the epileptiform discharges before (−2,700 s), during (−7,200 s), and after (−4,455 s) bumetanide application, which were recorded by four electrodes (numbers 54, 27, 22, 83) in **(A)**, corresponding to the epileptiform discharges in the DG, CA3, CA1, and subiculum, respectively. **(C)** The CA3- and Sub-origin epileptiform discharges in the rectangles (−1,200 s) in **(B)**, corresponding to the epileptiform discharges before (Control), during (Bumetanide), and after (Washout) bumetanide application. The red and blue rectangles represented the CA3- and Sub-origin epileptiform discharges, respectively. **(D)** The waveforms of the Sub-origin ictal-like discharges (IDs)/interictal-like discharges (IIDs), corresponding to the blue rectangles (−9 s) in **(C)** in an expanded time scale. **(E)** The waveforms of the CA3-origin IDs/IIDs, corresponding to the red rectangles (−30 s) in **(C)** in an expanded time scale.

To determine whether the Sub-origin epileptiform discharges were related with the NKCC1, we examined the effect of 10 µM bumetanide upon epileptiform discharges in the subiculum. At approximately 2,700–4,800 s (3,483.64 ± 154.49 s, *n* = 11) after the perfusion of low-Mg^2+^ ACSF, when 5–14 (9.09 ± 0.64, *n* = 11) stable discharge cycles occurred, bumetanide dissolved in low-Mg^2+^ ACSF was applied for 7,200 s (*n* = 11). Then, the drug was washed out by low-Mg^2+^ ACSF for about 3,200–7,200 s (4,841.55 ± 259.48 s, *n* = 11), until 4–10 (7.09 ± 0.52, *n* = 11) discharge cycles recovered in the slices. Figure 5B presents one example of the epileptiform discharges before (−2,700 s), during (−7,200 s), and after (−4,455 s) bumetanide application, and Figure 5C shows the CA3- and Sub-origin epileptiform discharges depicted in rectangles in Figure 5B. The epileptiform discharges in blue (Sub-origin) and red (CA3-origin) rectangles in Figure 5C are shown in an expanded time scale in Figures 5D,E, respectively.

Pooling all the data, during bumetanide application, the CA3-origin IDs in the HP disappeared, whereas the Sub-origin IDs, the CA3- and the Sub-origin IIDs continued to occur (*n* = 11). After the washout of bumetanide, the CA3-origin IDs recovered (*n* = 11), whereas the Sub-IDs only were only detected in some slices (*n* = 6).

Then, in order to obtain more quantitative information pertaining to the effect of bumetanide, we compared the parameters of the CA3- and Sub-origin epileptiform discharges before, during, and after bumetanide application.

**Figure 6:**
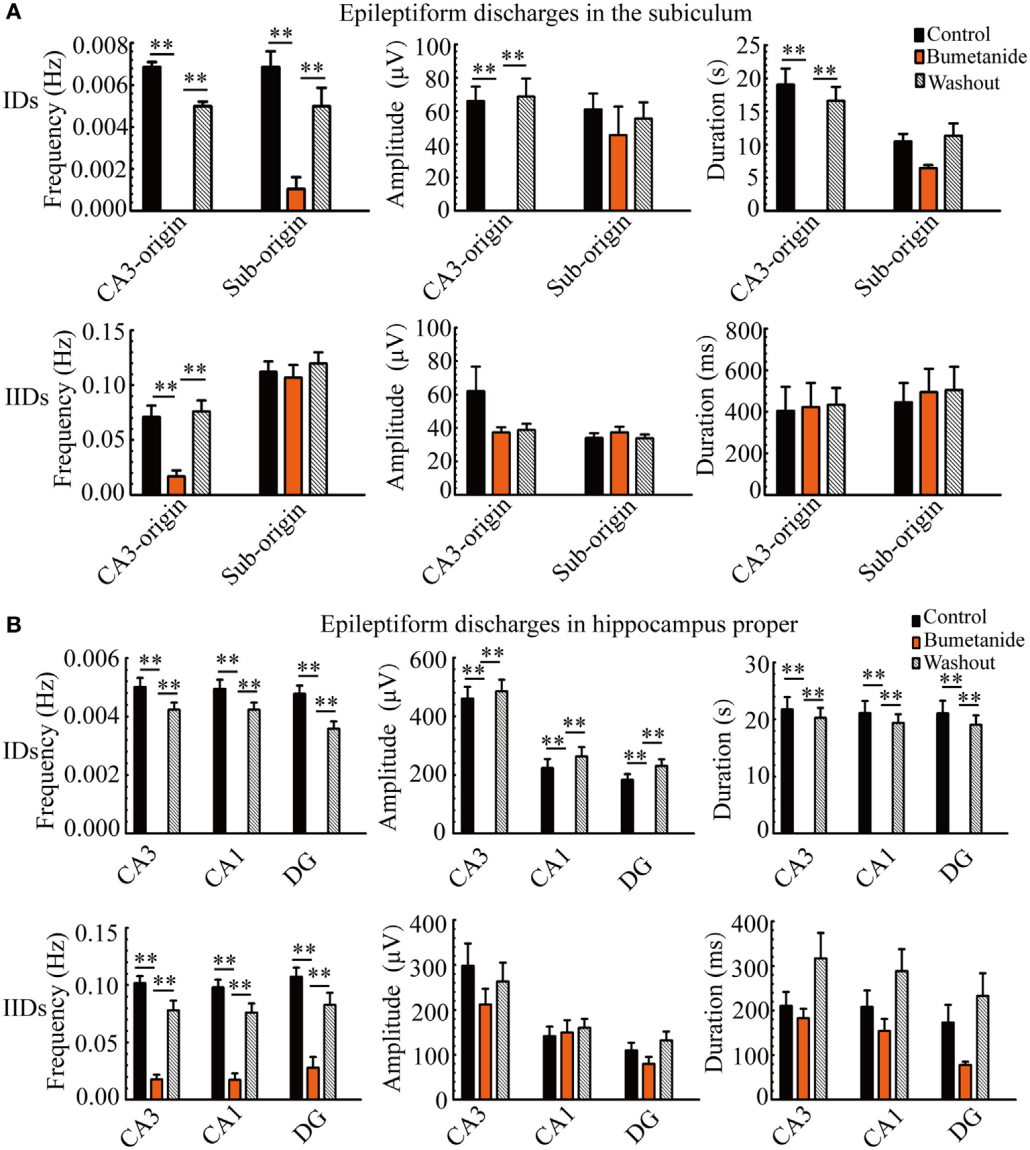
**The effect of bumetanide upon the parameters of epileptiform discharges**. **(A)** The effect of bumetanide upon the CA3- and Sub-origin epileptiform discharges in the subiculum. During bumetanide application, the CA3-origin ictal-like discharges (IDs) were reversely abolished. The frequencies of Sub-origin IDs and CA3-origin interictal-like discharges (IIDs) were significantly lower than those before/after bumetanide application [**P < 0.01, one-way analysis of variance (ANOVA), *n* = 11], whereas their amplitudes and durations were not significantly different (*P* > 0.05, one-way ANOVA, *n* = 11). During bumetanide application, the parameters of Sub-origin IIDs were not significantly different with those before/after bumetanide application (*P* > 0.05, one-way ANOVA, *n* = 11). **(B)** The effect of bumetanide upon the epileptiform discharges in the hippocampus proper (HP). The effect of bumetanide upon the IDs/IIDs in HP was similar to that upon the CA3-origin IDs/IIDs in the subiculum.

During bumetanide application, as shown in Figure 6A, in the subiculum, the CA3-origin IDs disappeared, while the Sub-origin IDs and CA3-origin IIDs continued to occur, and their frequencies were significantly lower than those before/after bumetanide application (**P < 0.01, one-way ANOVA, *n* = 11). However, their amplitudes and durations were not significantly changed. The Sub-origin IIDs were ongoing and their parameters were not significantly different with those before/after bumetanide application (*P* > 0.05, one-way ANOVA, *n* = 11).

Additionally, the effect of bumetanide upon the parameters of epileptiform discharges in the HP (CA3, CA1, DG) is shown in Figure 6B, which was analogous to that on the CA3-origin epileptiform discharges in the subiculum, as described above.

In addition, we had investigated the effect of 10 µM GABA_A_ receptor (GABA_A_R) antagonist bicuculline upon the ictogenesis in the subiculum and the HP. During bicuculline application, the CA3-origin IDs were abolished, while the Sub-origin IDs depressed instead of being abolished (Figures S2 and S3 in Supplementary Material).

These pharmacological results indicated that bumetanide/bicuculline had a different effect upon the CA3- and Sub-origin epileptiform discharges. They abolished the CA3-origin IDs, whereas the Sub-origin IDs continued to occur. This suggested that excitatory action of GABA resulting from NKCC1 played a less dominant role during ictogenesis in the subiculum than that in the HP. Moreover, during bumetanide/bicuculline application, when NKCC1/GABA_A_Rs were blocked while NMDARs were persistently activated, Sub-origin IDs continued to occur, therefore, the subicular ictogenesis might be related to glutamatergic excitation mediated by NMDARs.

**Figure 7:**
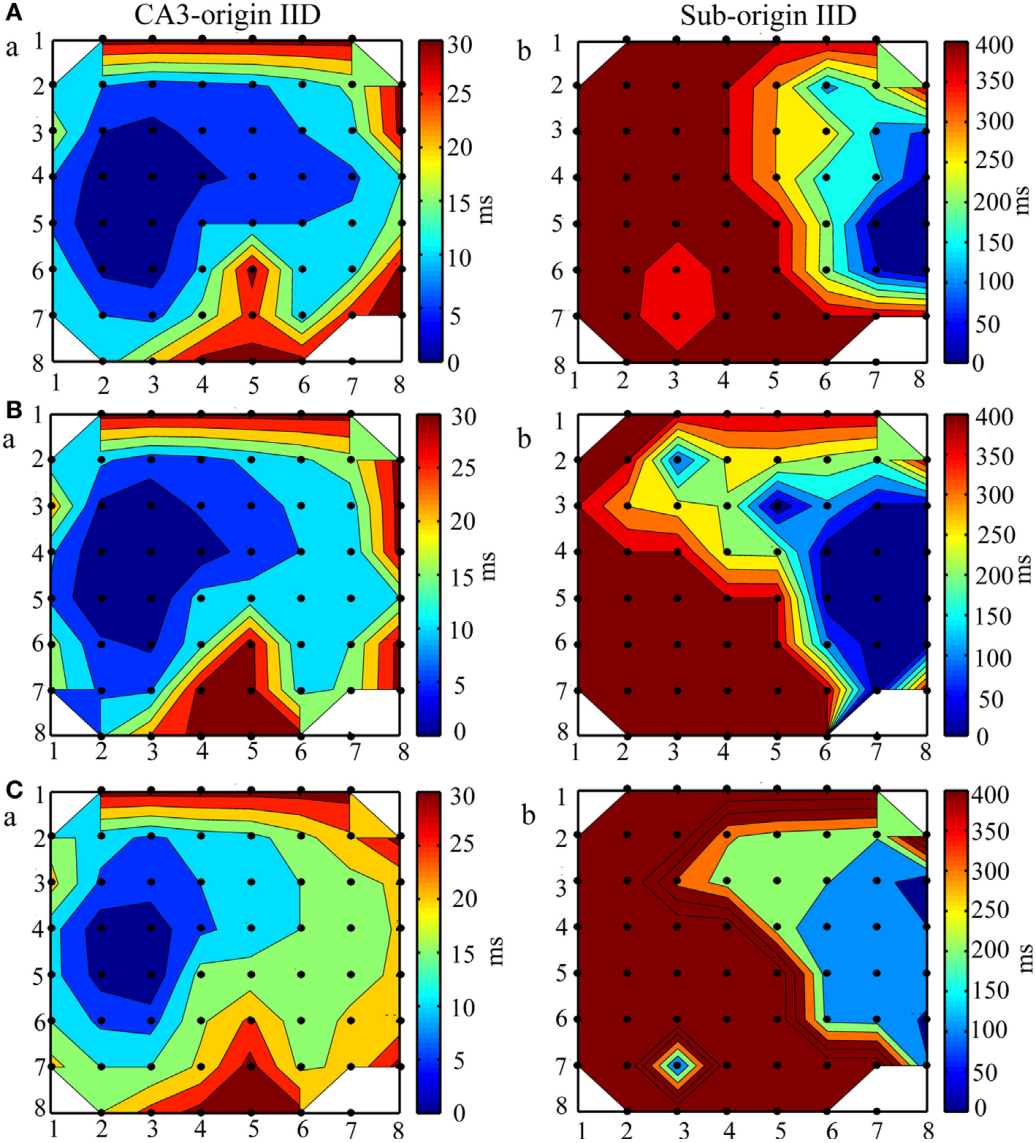
**The effect of bumetanide upon the initiation and propagation of interictal-like discharges (IIDs)**. **(A–C)** The contour plots for the averaged relative onset-time delays of IIDs before **(A)**, during **(B)**, and after **(C)** bumeatnide application in the slice shown in Figure 5A. The number of the electrodes (black dots) were denoted by the *x* and *y* coordinates. The color bar represents the relative onset-time delays. The different color in different regions symboled the initiate sites and propagation pathways. During bumetanide application, the IIDs had two initiations, CA3a/b (Ba) and the subiculum (Bb), the CA3-origin IIDs (Ba) initiated in the CA3a/b, propagated bidirectionally to CA1, subiculum (anterograde) and CA3c, DG (retrograde). The Sub-origin IIDs (Bb) initiated in the subiculum and did not propagate backward to the CA1. The two initiations and two corresponding propagation patterns of IIDs were not significantly different with those before/after bumetanide application.

Then, we evaluated whether bumetanide influenced the spatiotemporal dynamics of epileptiform discharges. As the CA3-origin IDs were abolished by bumetanide, we only investigated the effect of bumetanide on the initiations and propagation patterns of CA3- and Sub-origin IIDs. Taking the slice shown in Figure 5A as an example, the contour plots for the averaged onset-time delays of IIDs before (Figure 7A), during (Figure 7B), and after (Figure 7C) bumetanide application were compared. The initiations and propagation patterns of IIDs during bumetanide application were not significantly different with those before/after bumetanide application. The data from the other 10 slices also showed the similar results. The results revealed that bumetanide had no significant effect upon the spatiotemporal dynamics of epileptiform discharges.

### The Effect of d-AP5 upon Epileptiform Discharges

**Figure 8:**
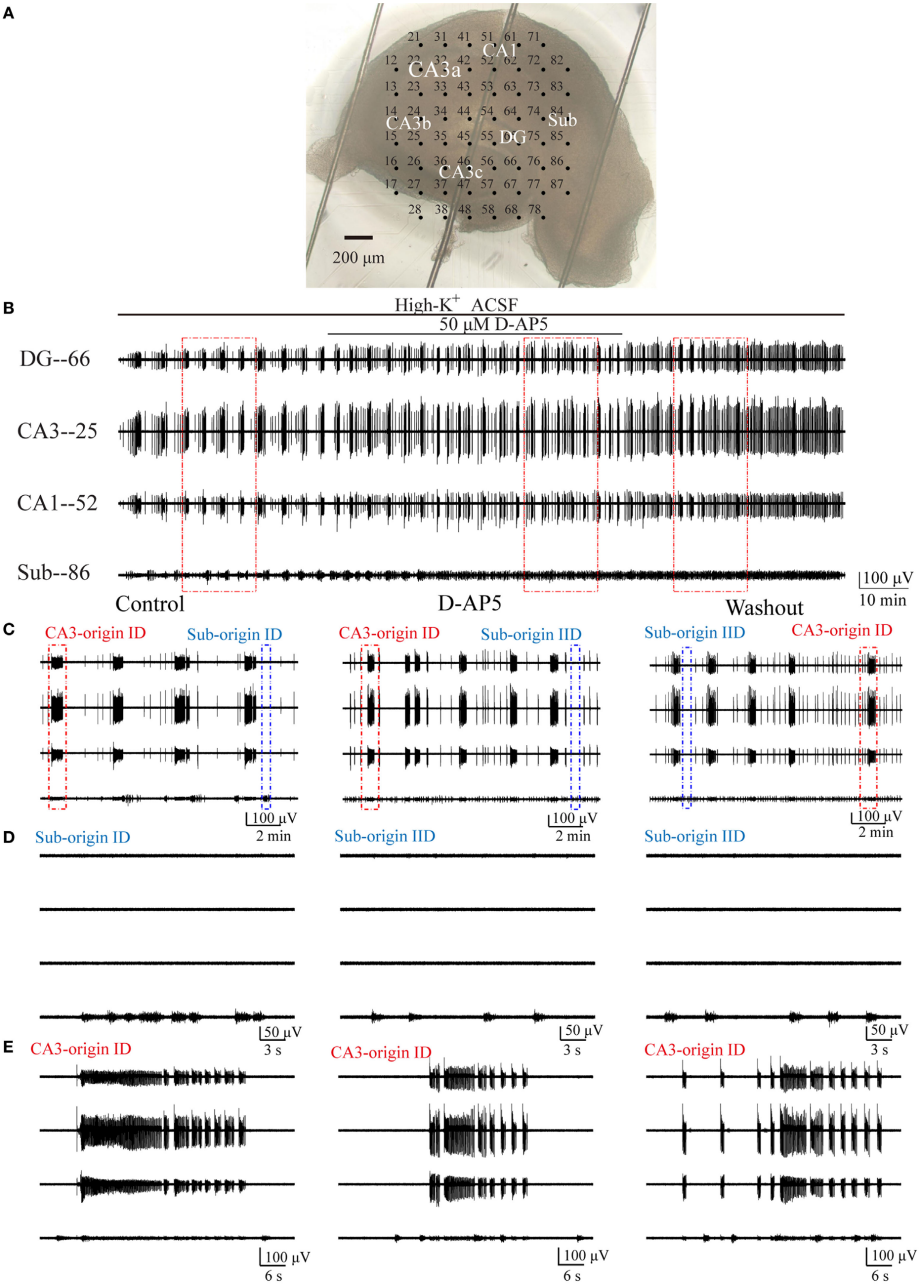
**Effect of d-AP5 upon the CA3- and Sub-origin epileptiform discharges**. **(A)** An example slice mounted on micro-electrode array. **(B)** The long-term display of the epileptiform discharges before (−2,560 s), during (−3,600 s), and after (−2,726 s) d-AP5 application, which were recorded by four electrodes (numbers 66, 25, 52, 86) in **(A)**, corresponding to the epileptiform discharges induced by high-K^+^ artificial cerebrospinal fluid in the DG, CA3, CA1, and subiculum, respectively. **(C)** The CA3- and Sub-origin epileptiform discharges in the rectangles (−900 s) in **(B)**, corresponding to the epileptiform discharges before (Control), during (d-AP5), and after (Washout) d-AP5 application. The red and blue rectangles represented the CA3- and Sub-origin epileptiform discharges, respectively. **(D)** The waveforms of the Sub-origin ictal-like discharges (IDs)/interictal-like discharge (IIDs), corresponding to the blue rectangles (−30 s) in **(C)** in an expanded time scale. **(E)** The waveforms of the CA3-origin IDs/IIDs, corresponding to the red rectangles (−60 s) in **(C)** in an expanded time scale.

According to the above results, we hypothesized that subicular ictogenesis might be related to glutamatergic excitation mediated by NMDARs. To verify this assumption, we tested the effect of 50 µM NMDAR antagonist d-AP5 upon epileptiform discharges in the subiculum. NMDARs were persistently activated in low-Mg^2+^ model, and it is inappropriate to apply NMDAR antagonist directly in low-Mg^2+^ model, so we applied d-AP5 in high-K^+^ model. At approximately 2,100–3,400 s (2,752 ± 151.28 s, *n* = 5) after the perfusion of high-K^+^ ACSF, when 5–10 (9.09 ± 0.64, *n* = 5) stable discharge cycles occurred, d-AP5 dissolved in high-K^+^ ACSF was applied for 2,700–3,900 s (3,490.4 ± 99.98 s, *n* = 5). Then, the drug was washed out by high-K^+^ ACSF for about 1,300–2,700 s (2,223.8 ± 123.66 s, *n* = 5), until 3–12 (8.09 ± 0.89, *n* = 5) discharge cycles recovered in the slices. An example slice was shown in Figure 8A, and Figure 8B presents examples of the epileptiform discharges before (−2,560 s), during (−3,600 s) and after (−2,726 s) d-AP5 application in this slice. Figure 8C shows the CA3- and Sub-origin epileptiform discharges depicted in rectangles in Figure 8B. The epileptiform discharges in blue (Sub-origin) and red (CA3-origin) rectangles in Figure 8C are shown in an expanded time scale in Figures 8D,E, respectively.

Pooling all the data (*n* = 5), during d-AP5 application, the Sub-origin IDs disappeared, whereas the Sub-origin IIDs and the CA3-origin IDs/IIDs continued to occur. After the washout of d-AP5, the Sub-origin IDs did not recover (*n* = 5).

Then, in order to obtain more quantitative information pertaining to the effect of d-AP5, we compared the parameters of CA3- and Sub-origin epileptiform discharges before, during, and after d-AP5 application.

**Figure 9:**
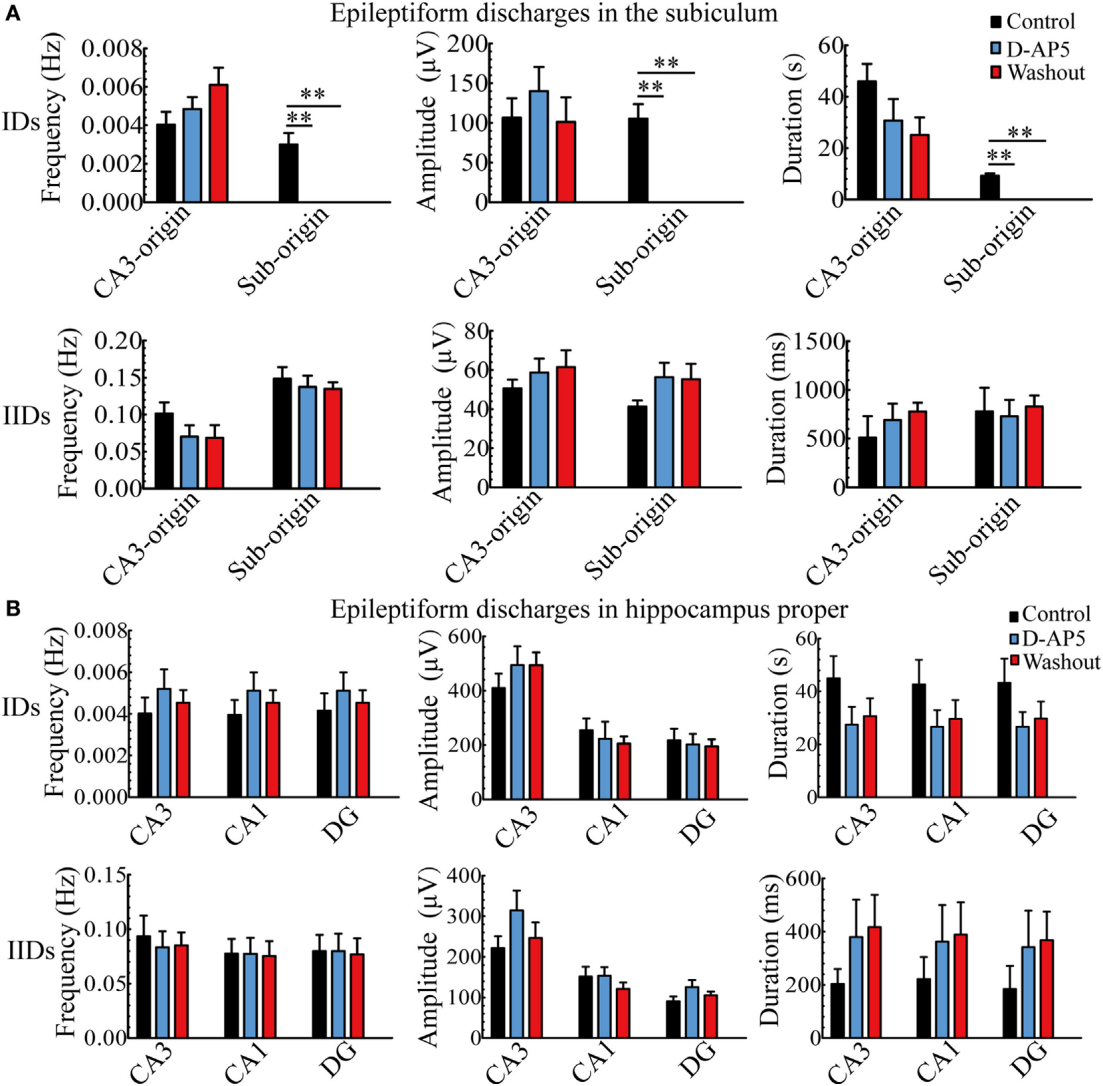
**The effect of d-AP5 upon the parameters of epileptiform discharges**. **(A)** The effect of d-AP5 upon the CA3- and Sub-origin epileptiform discharges in the subiculum. During d-AP5 application, the Sub-origin ictal-like discharges (IDs) were abolished; the frequencies, amplitudes, and durations of Sub-origin interictal-like discharge (IIDs) and CA3-origin IDs/IIDs were not significantly different with the Control/Washout group [*P* > 0.05, one-way analysis of variance (ANOVA), *n* = 5]. **(B)** The effect of d-AP5 upon the epileptiform discharges in the hippocampus proper (HP). The effect of d-AP5 upon the IDs/IIDs in the HP was similar to that upon the CA3-origin IDs/IIDs in the subiculum.

During d-AP5 application, as shown in Figure 9A, in the subiculum, the Sub-origin IDs disappeared, while the Sub-origin IIDs and CA3-origin IDs/IIDs continued to occur, and their frequencies, amplitudes, and durations were not significantly changed (*P* > 0.05, one-way ANOVA, *n* = 5).

Additionally, the effect of d-AP5 upon the parameters of epileptiform discharges in the HP (CA3, CA1, DG) is shown in Figure 9B, which was analogous to that on the CA3-origin epileptiform discharges in the subiculum, as described above.

These pharmacological results indicated that d-AP5 had a different effect upon the CA3- and Sub-origin epileptiform discharges. They abolished the Sub-origin IDs, whereas the CA3-origin IDs continued to occur. This suggested that glutamatergic excitation mediated by NMDARs played a more dominant role during ictogenesis in the subiculum than that in the HP. The subicular ictogenesis was related to glutamatergic excitation mediated by NMDARs.

### Protein Expression Levels in Subiculum and HP

#### Expression of NKCC1 in Subiculum and HP

**Figure 10:**
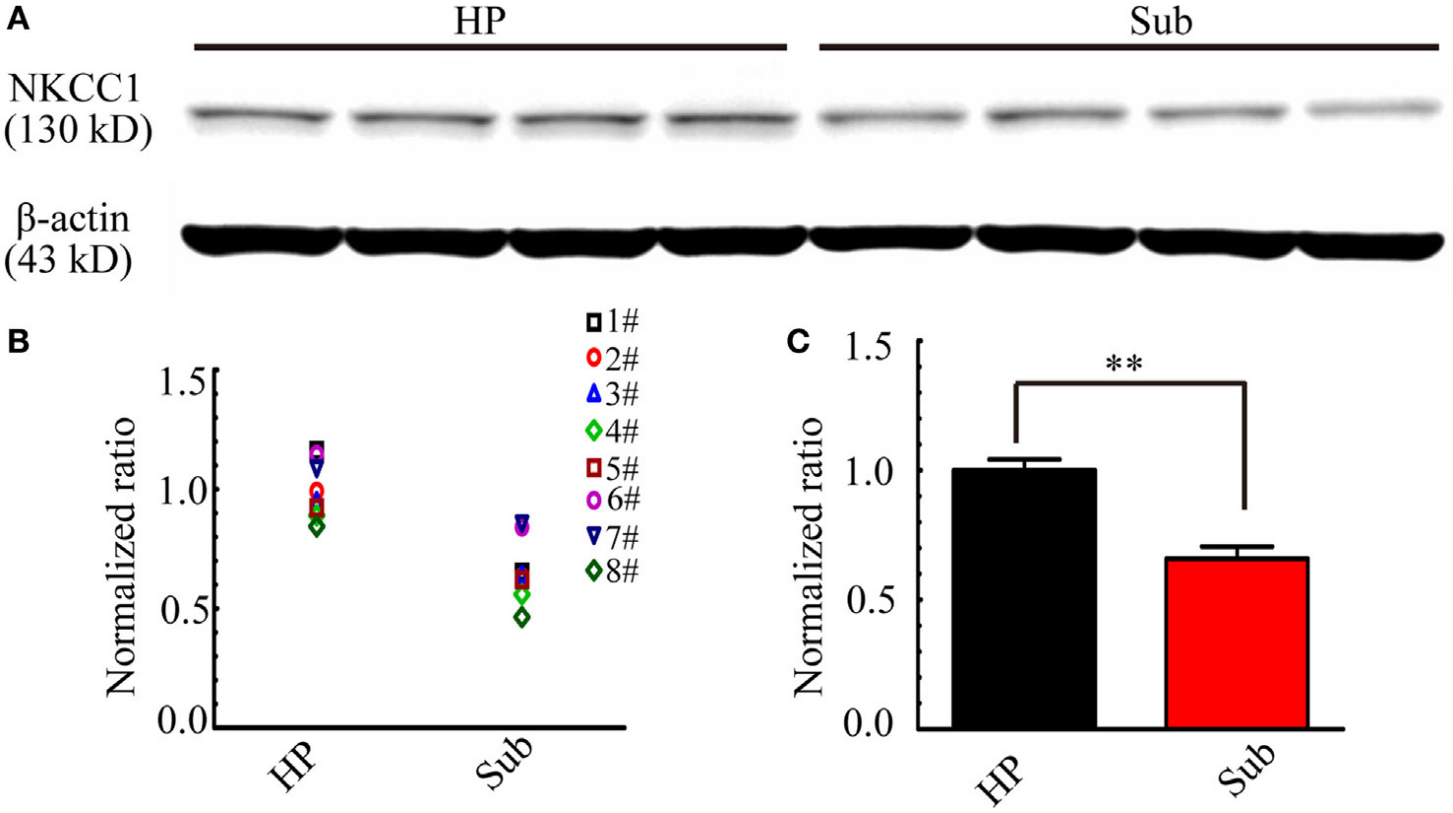
**Na^+^–K^+^–2Cl^−^ cotransporter 1 (NKCC1) expression level in the hippocampus proper (HP) and the subiculum**. **(A)** One example of Western blots of the NKCC1 in the HP and the subiculum (Sub). **(B,C)** Protein levels were quantified by densitometric signals obtained from Western blots, the level of NKCC1 protein in the subiculum were normalized to this protein level in the HP. The normalized protein levels in each mouse were displayed in **(B)**, and the averaged normalized protein levels of eight mice were displayed in **(C)**. The level of NKCC1 protein was significantly higher in the HP than that in the subiculum (**P < 0.01, paired *t*-test, *n* = 8).

The above results revealed that bumetanide was more effective in suppressing CA3-origin epileptiform discharges in the HP than Sub-origin epileptiform discharges in the subiculum. Bumetanide is a blocker of NKCC1, therefore, it is possible that the different effect of bumetanide upon CA3- and Sub-origin epileptiform discharges might be related to the differential expression levels of NKCC1 in the subiculum and HP. Thus, the expression of NKCC1 protein in the subiculum and HP was detected by Western blotting. Figure 10 shows one example of the Western blots (Figure 10A) and normalized expression level of NKCC1 protein (Figures 10B,C) in the subiculum and the HP. The results showed that NKCC1 protein expressed in the neonatal HP and the neonatal subiculum. The level of NKCC1 protein in the HP was significantly higher than that in the subiculum (**P < 0.01, paired *t*-test, *n* = 8). This result further explained why NKCC1 blocker, bumetanide, was more effective in suppressing the CA3-origin epileptiform discharges than the Sub-origin epileptiform discharges. In addition, these results suggested that NKCC1 expression is region-dependent, and GABAergic excitation resulting from NKCC1 was less dominant in the subiculum than that in the HP.

#### Expression of NR2A/B in Subiculum and HP

According to the results in electrophysiology, during d-AP5 application, the Sub-origin IDs were blocked, while the CA3-origin IDs continued to occur. d-AP5 is a blocker of NMDARs, therefore, it is possible that the different effect of d-AP5 upon CA3- and Sub-origin epileptiform discharges might be related to the differential expression levels of NMDARs in the subiculum and HP. The expression levels of NR2A and NR2B in the HP and the subiculum were further detected.

**Figure 11:**
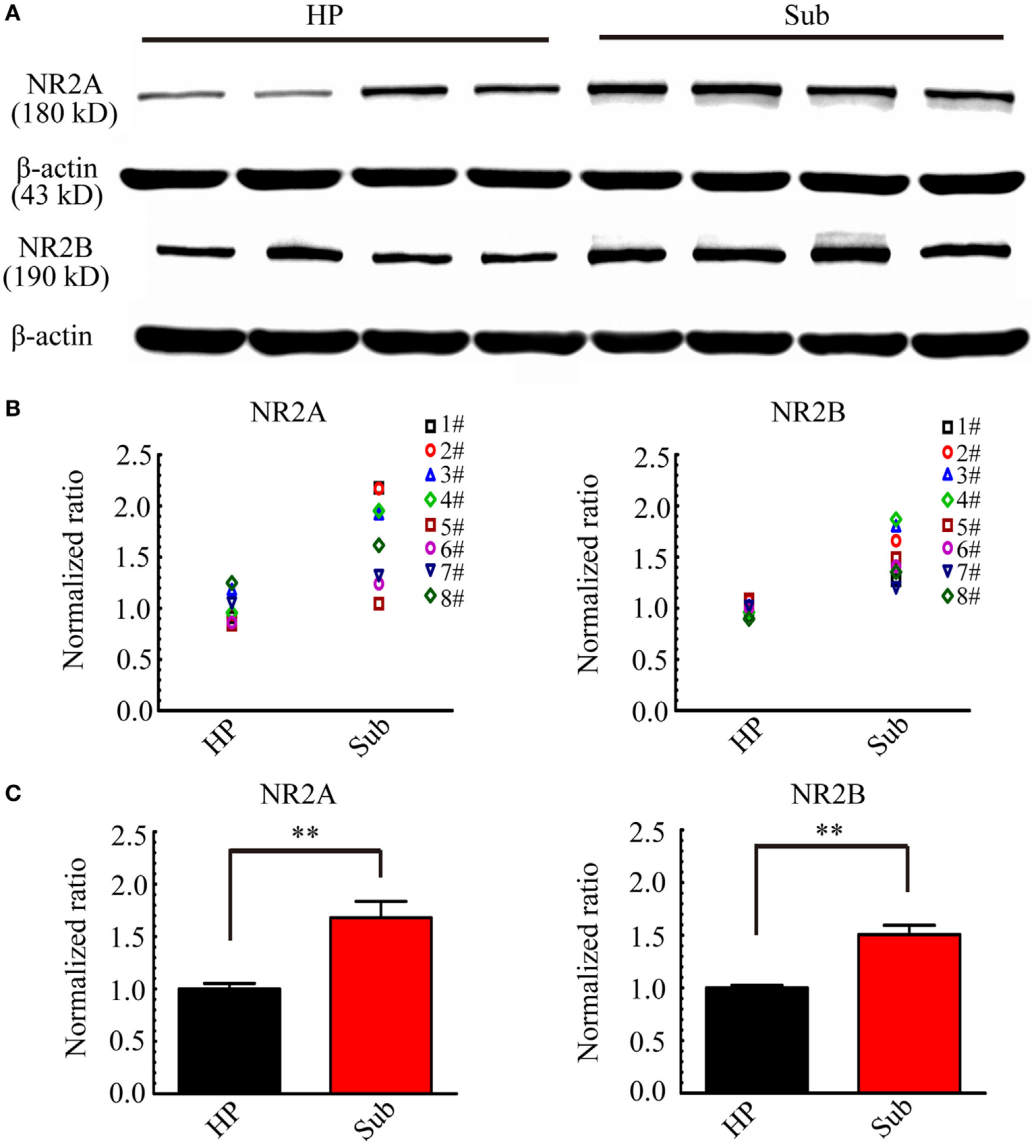
**NR2A and NR2B expression levels in the hippocampus proper (HP) and the subiculum**. **(A)** Examples of Western blots of the NR2A and NR2B in the HP and the subiculum (Sub). **(B,C)** Protein levels were quantified by densitometric signals obtained from Western blots. NR2A and NR2B protein levels in the subiculum were normalized to the corresponding protein level in the HP. The normalized protein levels in each mouse were displayed in **(B)**, and the averaged normalized protein levels of eight mice were displayed in **(C)**. The levels of NR2A and NR2B protein in HP were significantly lower than that in the subiculum (**P < 0.01, paired *t*-test, *n* = 8).

Figure 11 shows examples of the Western blots (Figure 11A) and normalized expression levels (Figures 11B,C) of NR2A/B protein in the HP and the subiculum. Our results showed that NR2A/B protein expressed in the neonatal HP and the neonatal subiculum. The levels of NR2A/B protein in the HP were lower than that in the subiculum (**P < 0.01, paired *t*-test, *n* = 8). The subiculum expressed abundant NMDARs, this result supported the electrophysiological deduction that the Sub-origin epileptiform discharges may be related to the glutamatergic excitation mediated by NMDARs.

## Discussion

Our data indicated that the subiculum was a potential site of ictogenesis in neonatal seizures and possessed similar seizure susceptibility to the HP. However, the mechanism underlying Sub-origin epileptiform discharges was different from that of the CA3-origin epileptiform discharges.

### Subiculum: A Potential Site of Ictogenesis for Neonatal Seizures

#### Pacemaker Cells and Axon Collaterals within the Subiculum

In the present study, the Sub-origin epileptiform discharges (IIDs and IDs) were discovered in neonatal hippocampal slices besides the CA3-origin epileptiform discharges (Figures 2 and 3). Consistent with previous studies, the CA3 of the HP was an ictogenesis site for neonatal seizures (37); moreover, our data revealed that the subiculum was a potential site of ictogenesis for neonatal seizures. This result was supported by the structural and electrophysiological characteristics of the neonatal subiculum. It has been demonstrated that the pyramidal cells and local collaterals of the subiculum in rodent animals become mature during the embryonic period, which means that the pyramidal cells and local collaterals in neonatal subiculum almost are similar to that in the adult subiculum (40–42). Studies on the morphology and electrophysiology of the adult subiculum have demonstrated that it possesses pacemaker cells and extensive local collaterals (7, 13). The subiculum possesses two types of pyramidal cells: burst firing cells and regular spiking cells, which can be classified according to their reactions to depolarizing current pulses (43). The burst firing cells serve as pacemaker cells for the generation of epileptiform discharges, as they have been found to be active in the absence of synchronous activity (7). The burst firing cells of the subiculum are located deep in the cell layer, and they give off one to three ascending axon collaterals extending to the mid-molecular layer, whereas the regular spiking cells are located superficially, and these cells have much broader axonal arbors in the cell layer (13). Furthermore, the subicular axons possess a high density of varicosities and extensions. Thereby, a pacemaker cell would activate its neighboring neurons depending on the collateralization of axons within the subiculum, thus synchronizing all regions of the subiculum. Additionally, it has been reported that an essential function of bursting cells is the amplification of neural signals (44), which facilitates the subiculum to work as an amplifier for information processing (43). In brief, the structural and physiological characteristics of the subiculum facilitate it to be an ictogenesis site for neonatal seizures.

#### Connections between the Subiculum and HP

In our experiments, the subiculum relayed the epileptiform discharges emanating from the HP, which initiated in CA3a/b, and propagated bidirectionally to CA1, subiculum (anterograde), to CA3c, and DG (retrograde) (Figure 2). This propagation pattern was supported by the initiating function of the CA3a/b neurons (6) and the synaptic connections among the sub-regions (DG, CA3, CA1, subiculum) (45–47). Amaral et al. revealed that all areas of the subiculum received CA1 projections (47). These CA1 to subiculum projections exist in a series of “nested loops,” which means that the cells in the proximal CA1 (near the CA3) projected to the distal subiculum (near the presubiculum), the cells in the middle part of CA1 projected to the middle portion of the subiculum, and the cells in the distal CA1 (near the subiculum) projected to the proximal subiculum (near the CA1) (48). The structural connections conducted the targeted transmission of epileptiform activities from the HP to the subiculum. As the major output station of the HP, the subiculum processes and amplifies a vast amount information from the HP (49). Previous electrophysiological studies *in vivo* and *in vitro* also showed that the electrical activities in CA1 of the HP propagated to the subiculum (50, 51).

In addition, our study showed that Sub-origin epileptiform discharges did not propagate backward to CA1. This was in line with a previous study showing that the CA1-subiculum pathway was unidirectional (51). The CA3-origin epileptiform discharges spread from the CA3 to CA1, then to the subiculum, relying upon preserved connectivity in the sub-regions. The rates of epileptiform discharges generated by the subiculum were less than that of the CA3, and CA1 was located between the subiculum and CA3, so it was much easier to follow the CA3 rhythm rather than the subiculum rhythm (12). However, another study showed that the epileptiform events generated in the subiculum could propagate backward to CA1 (12). This inconformity might be caused by the specific slices they used, which only contained the CA1 and subiculum regions. When CA1 is out of the control of CA3, it can relay the epileptiform activities initiating from the subiculum.

#### Extensive Interactions between the Subiculum and Other Brain Regions

In addition, previous neuropathological and electrophysiological studies on human and rat subiculum have revealed that the subiculum is able to relay information to downstream regions such as the subicular complex, the deep and superficial layers of EC, as well as various remote cortical and subcortical structures including the medial prefrontal cortex (PFC), retrosplenial cortex, nucleus accumbens, thalamus, and amygdala (52–58). A recent study on human brains also revealed that the subiculum possesses extensive collaterals projected to the brainstem (59). It is well known that a monosynaptic pathway exists between the subiculum and PFC, the midline thalamus connects the two regions through a polysynaptic path (60). Stimulation of the subiculum evoked the largest responses in the EC in cats (58), in the mediodorsal region of the thalamus, and the PFC in rats (60). Moreover, epileptiform activities propagating from the subiculum to EC assisted the synchronization of epileptic limbic networks, so abolishing this input would shorten the duration of ictal-like activities in the EC (61). Therefore, the epileptiform activities in the subiculum would propagate to downstream regions, and the subiculum would act as an epileptogenic focus to influence its downstream regions and thus play a pivotal role in maintaining the ictal-like activities in the limbic neural networks.

#### Similar Seizure Susceptibility in the Subiculum and HP

It has been reported that the latency to onset of epileptiform discharges in different areas could embody seizure susceptibilities in various areas (10). Our results showed that the latency to onset of the first epileptiform discharge was not significantly different when compared between the subiculum and the HP (Figure 3), which revealed that the seizure susceptibility in the subiculum was similar to that of the HP. In recent years, the role of the subiculum in adult TLE has attracted much attention (62), and research on TLE patients has found that the subiculum was the origin of IIDs in hippocampal slices (14). However, the role of the subiculum in neonatal seizures remained vague. Our study provided direct evidence that the subiculum not only served as a seizure focus during the neonatal period but also possessed the similar seizure susceptibility to the HP. Therefore, the subiculum should receive as much attention as the HP in future studies regarding neonatal seizures.

### Different Mechanisms Underlying CA3- and Sub-Origin Epileptiform Discharges

#### Unequal Role of GABAergic Excitation Resulting from NKCC1

During early postnatal period, GABA is strongly excitatory (16). Prior to glutamatergic innervation and at the time when glutamatergic synapses are “silent,” GABAergic innervation provides a major source for neuronal excitation (63). Activation of GABA_A_Rs is known to depolarize immature neurons in most regions of the CNS, including spinal cord (64), cerebellum (65), cortex (66), hippocampus (16), and olfactory bulb (67). However, along with development, the action of GABA gradually becomes less depolarizing and eventually transforms to be hyperpolarizing in the adult (16, 68). Previous research showed that the change in GABAergic action, from excitation to inhibition, occurred at postnatal day (P) 13.4 ± 0.4 in CA3 of rat hippocampal slices (8). In addition, GABAergic depolarization was reflected by an increase in intracellular [Ca^2+^]; a rapid and reversible increase in intracellular [Ca^2+^] occurred in P4–P10 hippocampal neurons in the exogenous applications of GABA, and no obvious elevation was observed in hippocampal neurons older than P13(69). Nevertheless, in the neurons of rat spinal cord and brain stem, GABAergic action was less excitatory at P4–P10 (70). These means that at the same age, GABAergic action was different in different regions.

In fact, GABAergic excitation results from the high expression of NKCC1 (71). Previous research has revealed that NKCC1 expression was different in the neurons of neonatal rat thalamus and neocortex (71). The EC and CA3 of the neonatal rat also showed different NKCC1 expression levels (10, 19); moreover, GABAergic action was different when compared between the ventroposterior thalamus and neocortex during the early development period (9). This implies that GABAergic excitation resulting from NKCC1 is unequal in different brain regions in the neonatal period.

During neonatal period, NKCC1 facilitates the accumulation of intracellular Cl^−^ and results in a depolarized E_Cl_ (71), so the activation of Cl^−^ permeable GABA_A_Rs excites neurons (17, 69, 72). Bumetanide is a blocker of NKCC1 at low doses (2–10 µM) (73). It reduces intracellular Cl^−^ accumulation and results in a lower concentration of intracellular Cl^−^, and shifts E_Cl_ negative. Therefore, in the presence of bumetanide, the activation of Cl^−^ permeable GABA_A_Rs would induce Cl^−^ influx and lead to a hyperpolarizing effect (11, 74, 75). The anticonvulsant action of bumetanide in immature animal models was achieved by preventing the accumulation of intracellular Cl^−^, and switched the excitatory action of GABA into a hyperpolarizing effect (11, 18). Consistent with previous studies (11, 76), our pharmacological experiments showed that bumetanide reversely abolished the CA3-origin IDs and depressed the CA3-origin IIDs in the HP in low-Mg^2+^ model (Figures 5 and 6). This suggested that, in low-Mg^2+^ model, the E_Cl_ in neonatal CA3 neurons was depolarized, and bumetanide shifted E_Cl_ negative and switched the GABAergic excitation mediated by GABA_A_Rs into inhibition, and the IDs were suppressed.

However, the research conducted by Kilb et al. showed that bumetanide did not suppress ictal activity in low-Mg^2+^ model (77), this discrepancy might result from two reasons: firstly, it might result from different animal age and gender we used. In our experiment, we used P8–P10 male mice, while in the research conducted by Kilb et al., P4–P7 mice were used. Their animal age were not identical with our animals, and they also did not distinguish the animal gender. Previous research has showed that NKCC1 expression was region- and age-dependent (10), and it is also sexually dimorphic in the hippocampus of neonatal rats: in males, NKCC1 expression was constant during the first week, peaking almost three-fold higher than the initial level at P9, and the level of NKCC1 was higher at P8–P10 than that at P4–P7 in the hippocampus; while in the females, NKCC1 expression was constant during P1–P15 (19). Bumetanide is the blocker of NKCC1, therefore, its antiepileptic action was related to the level of NKCC1 expression. It is reasonable to infer that the effect of bumetanide might be more significant in P8–10 male mouse hippocampus than P4–P7 mouse hippocampus. Secondly, the discrepancy might be explained by differences in functional connectivity between both tissue preparations. In our experiment, we used hippocampal slices, while in the research conducted by Kilb et al., intact hippocampal preparations were used.

In addition, during bumetanide application, the Sub-origin IDs were depressed instead of being abolished, and Sub-origin IIDs were not affected (Figures 5 and 6). It is reasonable to infer that E_Cl_ in the subicular neurons was also depolarized, bumetanide shift the E_Cl_ in subicular neurons negative, but GABAergic excitation was less dominant during the subicular ictogenesis, so when NKCC1 was blocked and GABAergic excitation was blocked, the Sub-origin IDs still occurred. Additionally, during bicuculline application, GABA_A_Rs were blocked while NMDARs were activated, the CA3-origin IDs were abolished, while the Sub-origin IDs still occurred (Figures S2 and S3 in Supplementary Material). Previous research has reported that, in the neonatal period, GABA was strongly excitatory and GABA excitation mediated by GABA_A_Rs contributes to the initiation of IDs in the developing hippocampus (5, 8, 16, 78). In the adult rat, GABA excitation mediated by GABA_A_Rs switched to inhibition, so the ictal activity cannot be induced in hippocampal slices (16). Bicuculline is the antagonist of GABA_A_Rs, so when GABA_A_Rs were blocked by bicuculline, the GABA excitation mediated by GABA_A_Rs was blocked, and the IDs resulting from GABA excitation mediated by GABA_A_Rs also disappeared (Figures S2 and S3 in Supplementary Material). Our result was consistent with the previous research that bicuculline abolished the ictal discharges in the hippocampal slices from P5–P20 rats (16). Unlike the CA3-origin IDs, the Sub-origin IDs cannot be abolished by bicuculline, this result further provided evidence that GABAergic excitation played a less dominant role during ictogenesis in the subiculum than that in the HP.

In addition, our Western blotting analysis showed that in P8–P10 mice, the level of NKCC1 protein was higher in the HP than that in the subiculum (Figure 8). This result explained why the antiepileptic action of bumetanide was more effective in the HP than that in the subiculum. Meanwhile, these data was consistent with the electrophysiological inference that excitatory action of GABA resulting from NKCC1 played a less dominant role during ictogenesis in the subiculum than that in the HP.

Additionally, in the early postnatal period, giant depolarizing potential (GDPs), generated by a population of synchronized neurons, and was mediated by GABAergic excitation. GDPs were frequently observed during the first postnatal week of rat hippocampal neurons (67, 79–81); they were less frequent between P9–P12 and disappeared after P12 (82, 83). In our experiment, we used the P8–P10 mice and multi-electrode recording techniques (an extracellular recording), and GDPs were not discovered in the slices.

#### Unequal Role of Glutamatergic Excitation Mediated by NMDARs

During early neonatal period, glutamatergic excitation is too weak to support the ictal epileptiform activity (16). Along with development, the percentage of “silent” glutamatergic synapses progressively decreased, the glutamatergic excitation is stronger and stronger, and it gradually become the major source for neuronal excitation. Additionally, glutamatergic synaptic transmission is reported to be mediated by pure NMDARs during the second and third postnatal week in hippocampus (84). There is some evidence to suggest that NR2A/B-containing NMDARs show differentially regional expression levels in neonatal period. In P7–P12 rat brain, the level of NR2A mRNA was higher in the cortex than that in the thalamic nuclei and cerebellum, the level of NR2B mRNA was higher in the cortex than that in the striatum, septum, thalamic nuclei and cerebellum (85). This means that in the same period, glutamatergic excitation mediated by NMDARs was unequal in different regions.

Our electrophysiological experiments showed that during d-AP5 application, when NMDARs were blocked, the Sub-origin IDs disappeared, while the CA3-origin IDs still occurred (Figures 8 and 9). Therefore, it was logical to deduce that, in P8–P10 mice, glutamatergic excitation mediated by NMDARs played a more dominant role during ictogenesis in the subiculum than that in the HP, and subicular ictogenesis might be related to the glutamatergic excitation mediated by NMDARs. In addition, our Western blotting analysis showed that the NR2A/B protein levels were higher in the subiculum than that in the HP (Figure 11). This provided additional evidence to support the conclusion that glutamatergic excitation might be more dominant during ictogensis in the subiculum than that in the HP, and subicular ictogenesis was related to the glutamatergic excitation mediated by NMDARs.

In conclusion, our current research revealed that the subiculum was a potential site of ictogenesis in neonatal seizure models, and that it possessed similar seizure susceptibility to that of the HP. However, the underlying mechanism during ictogenesis in the subiculum was different from that in the HP. GABAergic excitation resulting from NKCC1 played a less dominant role during ictogenesis in the subiculum than that in the HP, while glutamatergic excitation mediated by NMDARs played a more dominant role during ictogenesis in the subiculum than that in the HP. Subicular ictogenesis was related to the glutamatergic excitation mediated by NMDARs. Consequently, for neonates suffering from seizures, if the subiculum was the seizure initiation site, bumetanide might be a less effective therapeutic option, and that drugs targeted to block NMDARs might be an effective treatment.

## Ethics Statement

This study was carried out in accordance with the recommendations of Laboratory animal research program, Shanghai Jiao Tong University. The protocol was approved by the Ethic Committee, School of Biomedical Engineering, Shanghai Jiao Tong University.

## Author Contributions

X-XW performed the experiments, participated in the design of the study, analyzed the data, and wrote the paper. Y-HL analyzed the statistical data. H-QG and P-JL contributed with the laboratory support and revised the manuscript. P-MZ and Q-CL conceived the study, participated in its design, coordination, and revised the manuscript.

## Conflict of Interest Statement

The authors declare that the research was conducted in the absence of any commercial or financial relationships that could be construed as a potential conflict of interest.

## Supplementary Material

Figure S1 **Initiation and propagation pattern of IDs/interictal-like discharge (IIDs) in the other 20 slices**. **(A)** Images of the hippocampal slices mounted on micro-electrode array (*n* = 20). **(B)** Two types of contour plots for the averaged relative onset-time delays of all the IDs (a,b) and IIDs (c,d) in the corresponding slice shown in A. The number of the electrodes was denoted by the *X* and *Y* coordinates. The color bar represents the relative onset-time delays. The different color in different regions symboled the initiate sites and propagation pathways. In each slice, the epileptiform discharges (IDs/IIDs) had two initiations (dark blue regions), CA3a/b (Ba, Bc) and the subiculum (Bb, Bd). The CA3-origin epileptiform discharges (Ba, Bc) initiated in CA3a/b and propagated bidirectionally to the CA1, subiculum (anterograde), to the CA3c, DG (retrograde). The Sub-origin epileptiform discharges (Bb, Bd) initiated in the subiculum and did not propagate backward to the CA1.

Figure S2 **Effect of bicuculline upon the CA3- and Sub-origin epileptiform discharges**. **(A)** An example slice mounted on micro-electrode array. **(B)** The long-term display of the epileptiform discharges before (−3,360 s), during (−5,000 s), and after (−2,020 s) bicuculline application, which were recorded by four electrodes (numbers 54, 25, 42, 83) in **(A)**, corresponding to the epileptiform discharges in the DG, CA3, CA1, and subiculum, respectively. **(C)** The CA3- and Sub-origin epileptiform discharges in the rectangles (−900 s) in **(B)**, corresponding to the epileptiform discharges before (Control), during (Bicuculline) and after (Washout) bicuculline application. The red and blue rectangles represented the CA3- and Sub-origin epileptiform discharges respectively. **(D)** The waveforms of the Sub-origin IDs/interictal-like discharges (IIDs), corresponding to the blue rectangles (−9 s) in **(C)** in an expanded time scale. **(E)** The waveforms of the CA3-origin IDs/IIDs, corresponding to the red rectangles (−30 s) in **(C)** in an expanded time scale.

Figure S3 **Effect of bicuculline upon parameters of epileptiform discharges**. **(A)** Effect of bicuculline upon the CA3- and Sub-origin epileptiform discharges in the subiculum. During bicuculline application, the CA3-origin IDs were reversely abolished. The frequency of Sub-origin IDs was significantly lower than those before/after bicuculline application [***P* < 0.01, one-way analysis of variance (ANOVA), *n* = 9], whereas the amplitude and duration of Sub-origin IDs were not significantly different (*P* > 0.05, one-way ANOVA, *n* = 11). During bicuculline application, the parameters of CA3- and Sub-origin interictal-like discharges (IIDs) were not significantly different with those before/after bicuculline application (*P* > 0.05, one-way ANOVA, *n* = 11). **(B)** The effect of bicuculline upon the epileptiform discharges in the hippocampus proper (HP). The effect of bicuculline upon the IDs/IIDs in HP was similar to that upon the CA3-origin IDs/IIDs in the subiculum.
