# Supplementary figures and images for: The Subiculum: A Potential Site of Ictogenesis in a Neonatal Seizure Model
Source: Front Neurol. 2017 Apr 20;8:147. doi: 10.3389/fneur.2017.00147 (PMC5397469; doi:10.3389/fneur.2017.00147)

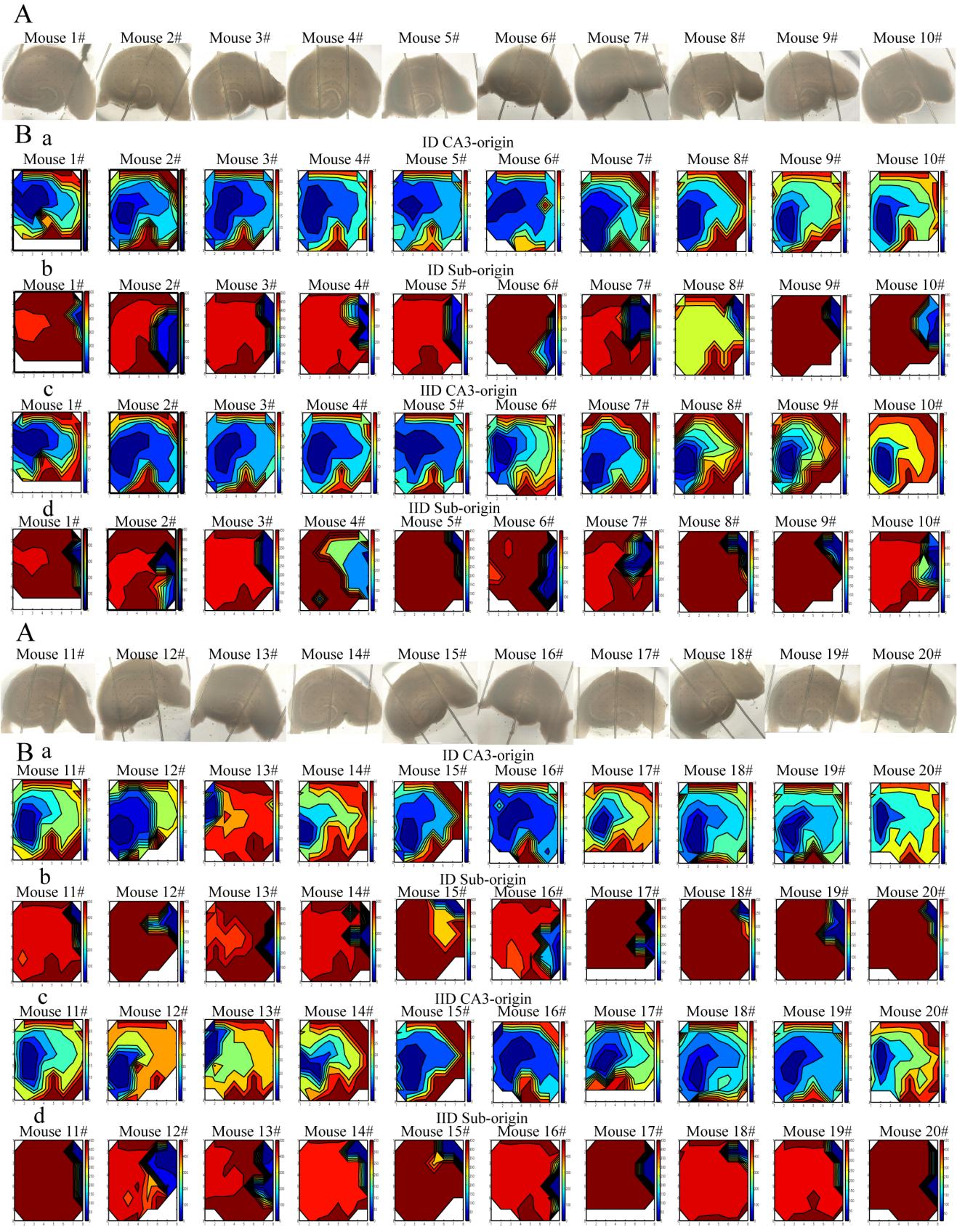

Supplement: Figure S1 — Initiation and propagation pattern of IDs/interictal-like discharge (IIDs) in the other 20 slices. (A) Images of the hippocampal slices mounted on micro-electrode array (n = 20). (B) Two types of contour plots for the averaged relative onset-time delays of all the IDs (a,b) and IIDs (c,d) in the corresponding slice shown in A. The number of the electrodes was denoted by the X and Y coordinates. The color bar represents the relative onset-time delays. The different color in different regions symboled the initiate sites and propagation pathways. In each slice, the epileptiform discharges (IDs/IIDs) had two initiations (dark blue regions), CA3a/b (Ba, Bc) and the subiculum (Bb, Bd). The CA3-origin epileptiform discharges (Ba, Bc) initiated in CA3a/b and propagated bidirectionally to the CA1, subiculum (anterograde), to the CA3c, DG (retrograde). The Sub-origin epileptiform discharges (Bb, Bd) initiated in the subiculum and did not propagate backward to the CA1. [file Image_1.PDF]

A

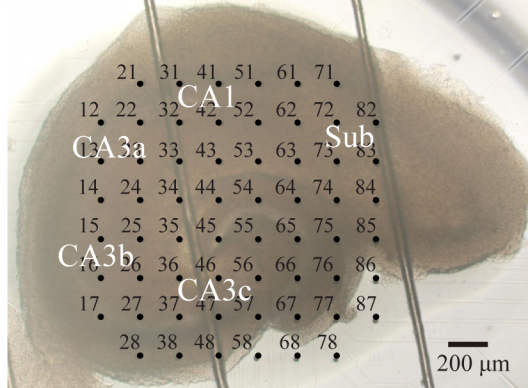

B

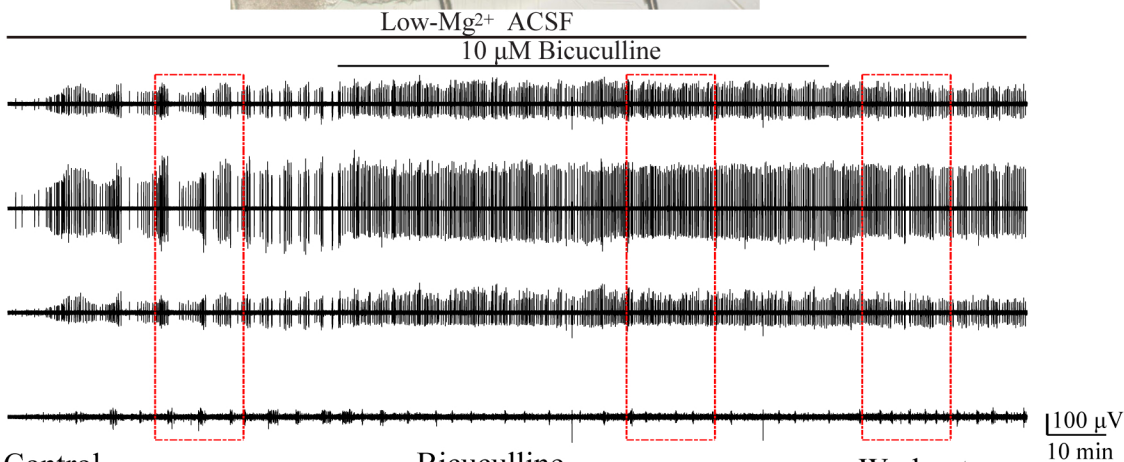

C

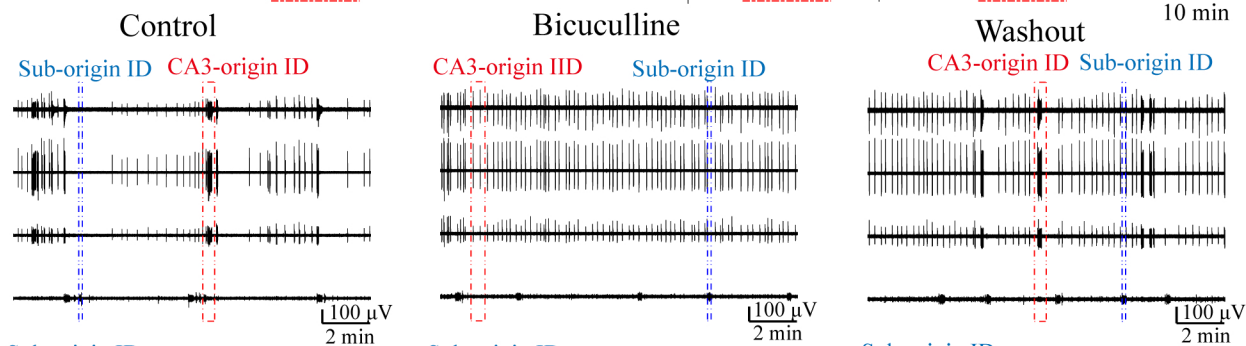

D

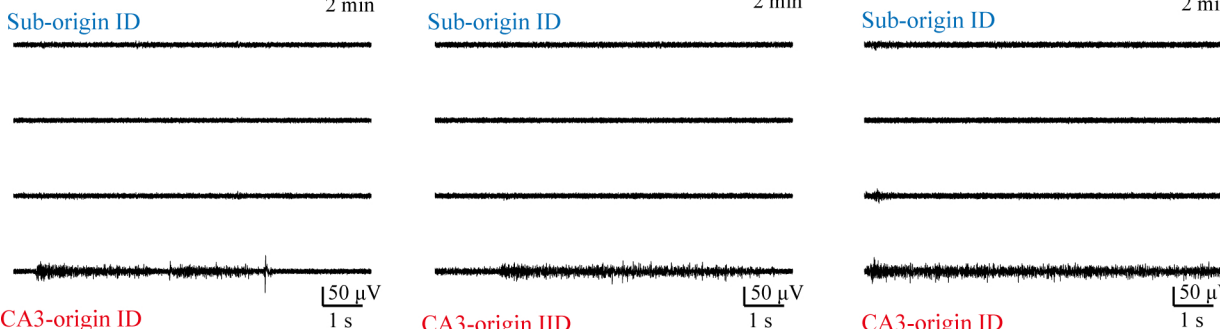

E

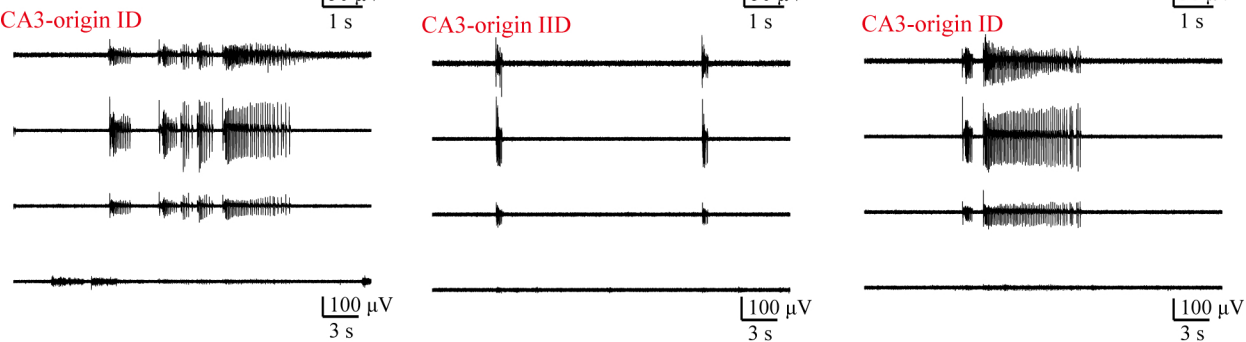

Supplement: Figure S2 — Effect of bicuculline upon the CA3- and Sub-origin epileptiform discharges. (A) An example slice mounted on micro-electrode array. (B) The long-term display of the epileptiform discharges before (−3,360 s), during (−5,000 s), and after (−2,020 s) bicuculline application, which were recorded by four electrodes (numbers 54, 25, 42, 83) in (A), corresponding to the epileptiform discharges in the DG, CA3, CA1, and subiculum, respectively. (C) The CA3- and Sub-origin epileptiform discharges in the rectangles (−900 s) in (B), corresponding to the epileptiform discharges before (Control), during (Bicuculline) and after (Washout) bicuculline application. The red and blue rectangles represented the CA3- and Sub-origin epileptiform discharges respectively. (D) The waveforms of the Sub-origin IDs/interictal-like discharges (IIDs), corresponding to the blue rectangles (−9 s) in (C) in an expanded time scale. (E) The waveforms of the CA3-origin IDs/IIDs, corresponding to the red rectangles (−30 s) in (C) in an expanded time scale. [file Image_2.PDF]
