# Supplementary material for: The Subiculum: A Potential Site of Ictogenesis in a Neonatal Seizure Model
Source: Front Neurol. 2017 Apr 20;8:147. doi: 10.3389/fneur.2017.00147 (PMC5397469; doi:10.3389/fneur.2017.00147)
Supplement: Figure S3 — Effect of bicuculline upon parameters of epileptiform discharges. (A) Effect of bicuculline upon the CA3- and Sub-origin epileptiform discharges in the subiculum. During bicuculline application, the CA3-origin IDs were reversely abolished. The frequency of Sub-origin IDs was significantly lower than those before/after bicuculline application [**P < 0.01, one-way analysis of variance (ANOVA), n = 9], whereas the amplitude and duration of Sub-origin IDs were not significantly different (P > 0.05, one-way ANOVA, n = 11). During bicuculline application, the parameters of CA3- and Sub-origin interictal-like discharges (IIDs) were not significantly different with those before/after bicuculline application (P > 0.05, one-way ANOVA, n = 11). (B) The effect of bicuculline upon the epileptiform discharges in the hippocampus proper (HP). The effect of bicuculline upon the IDs/IIDs in HP was similar to that upon the CA3-origin IDs/IIDs in the subiculum. [file Image_3.PDF]

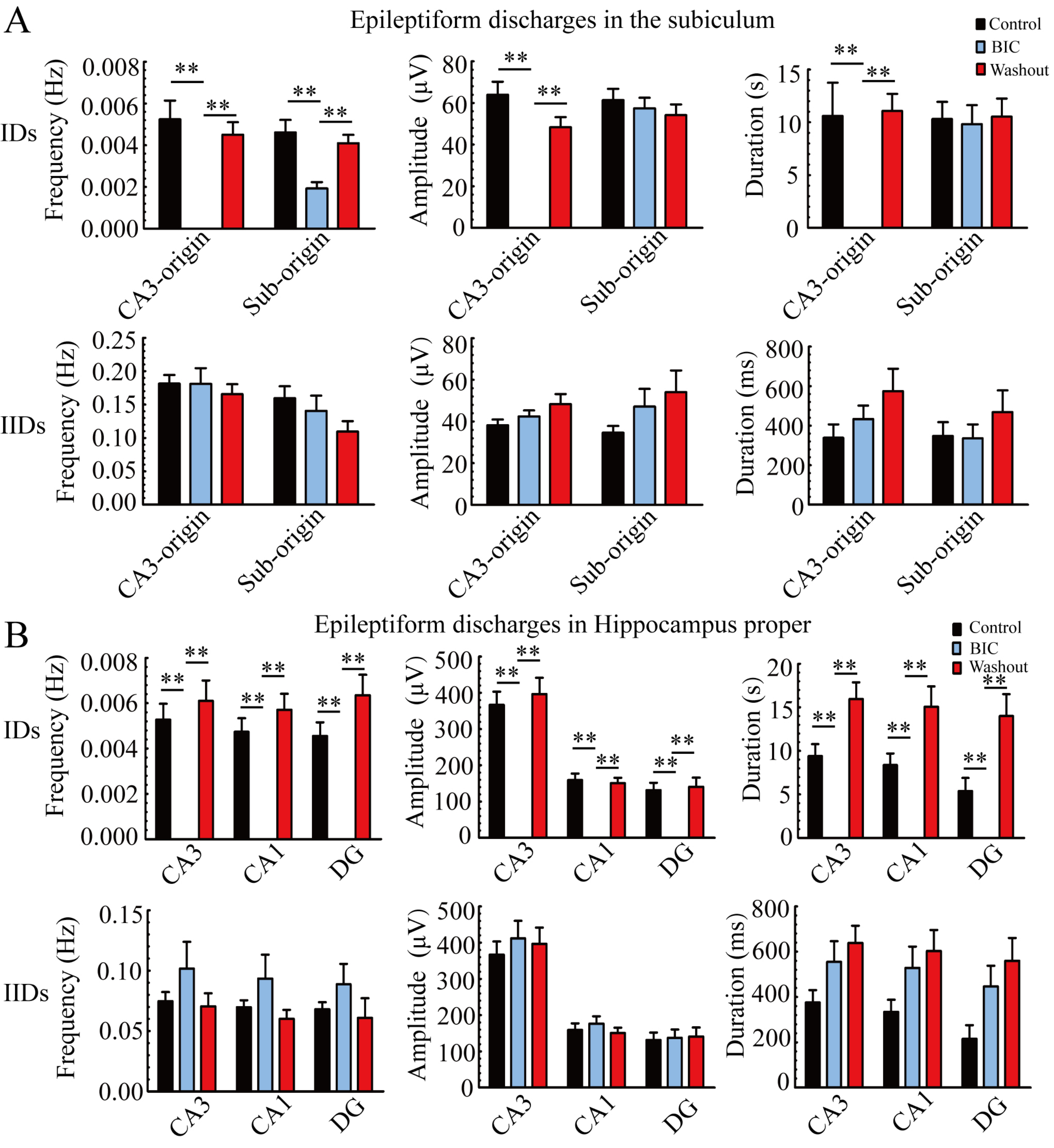
A
Epileptiform discharges in the subiculum
Control
BIC
Washout
IDs
Frequency (Hz)
Amplitude (μV)
Duration (s)
CA3-origin
Sub-origin
IIDs
Duration (ms)
B
Epileptiform discharges in Hippocampus proper
CA3
CA1
DG
